# Distinct signaling signatures drive compensatory proliferation via S-phase acceleration

**DOI:** 10.1371/journal.pgen.1010516

**Published:** 2022-12-15

**Authors:** Carlo Crucianelli, Janhvi Jaiswal, Ananthakrishnan Vijayakumar Maya, Liyne Nogay, Andrea Cosolo, Isabelle Grass, Anne-Kathrin Classen

**Affiliations:** 1 Hilde-Mangold-Haus, University of Freiburg, Freiburg, Germany; 2 Faculty of Biology, University of Freiburg, Freiburg, Germany; 3 Spemann Graduate School of Biology and Medicine (SGBM), University of Freiburg, Freiburg, Germany; 4 International Max Planck Research School for Immunobiology, Epigenetics, and Metabolism, Freiburg, Germany; 5 BIOSS Centre for Biological Signalling Studies, University of Freiburg, Freiburg, Germany; 6 CIBSS Centre for Integrative Biological Signalling Studies, University of Freiburg, Freiburg, Germany; Instituto Leloir, ARGENTINA

## Abstract

Regeneration relies on cell proliferation to restore damaged tissues. Multiple signaling pathways activated by local or paracrine cues have been identified to promote regenerative proliferation. How different types of tissue damage may activate distinct signaling pathways and how these differences converge on regenerative proliferation is less well defined. To better understand how tissue damage and proliferative signals are integrated during regeneration, we investigate models of compensatory proliferation in *Drosophila* imaginal discs. We find that compensatory proliferation is associated with a unique cell cycle profile, which is characterized by short G1 and G2 phases and, surprisingly, by acceleration of the S-phase. S-phase acceleration can be induced by two distinct signaling signatures, aligning with inflammatory and non-inflammatory tissue damage. Specifically, non-autonomous activation of JAK/STAT and Myc in response to inflammatory damage, or local activation of Ras/ERK and Hippo/Yki in response to elevated cell death, promote accelerated nucleotide incorporation during S-phase. This previously unappreciated convergence of different damaging insults on the same regenerative cell cycle program reconciles previous conflicting observations on proliferative signaling in different tissue regeneration and tumor models.

## Author summary

Regeneration requires high levels of cell proliferation to restore the physiological function a damaged tissue. This can either be achieved by allowing more cells to enter the cell cycle, or by accelerating cell cycle progression. In addition, the spatio-temporal dynamics of proliferation need to account for differences in the types and degree of damage. How different tissue damage environments, proliferative signals and cell cycle controls are integrated to drive tissue regeneration is little understood. We address this open question in regenerating *Drosophila* imaginal discs. We demonstrate that inflammatory and non-inflammatory damage activate distinct proliferative signaling pathways, which accelerate cell cycle progression via reducing gap phase length. Despite the risk of inducing replicative stress and compromising genome integrity, we find that nucleotide incorporation during DNA replication is strongly and safely accelerated, thereby also reducing S-phases length. Our work thus provides an unprecedented perspective on the convergence of different damage and signaling environments on the same regenerative cell cycle program.

## Introduction

Tissue regeneration in many systems relies on the induction of cell proliferation in stem cells [1] or other tissue-resident cell types [2] to restore the damaged tissue. The signaling pathways that regulated regenerative proliferation have been extensively explored [3, 4]. Yet how different types of tissue damage may activate distinct signaling pathways and how these different signals converge on regenerative proliferation is less well defined.

Epithelia are tissues with high regenerative capacity. Epithelia cover the surfaces of many organs and execute one core function: to act as barrier between internal and external environments [5]. This essential function may be challenged by different types of damage, such as toxins, wounding and infection. Mild insults may just induce elevated cell death. Cell death in epithelia is highly regulated to maintain junctional integrity and barrier function, even at decreasing cell density [6–8]. Thus, regenerative proliferation needs to respond to a geometrically altered environment (reduced cell density) which still performs normal epithelial functions. However, if the rate of cell death exceeds regenerative proliferation, barrier function breaks down [9]. Similarly, physical wounding or pathological processes strongly disrupt epithelial barrier integrity. In response, inflammation and associated cellular responses, which strongly alter cell behavior, are activated. These include upregulation of cytokine signaling, ROS production, migratory behaviors and even senescence, all geared towards preventing infection and efficiently closing the wound to restore the barrier [10–12]. Whether these different inflammatory and non-inflammatory scenarios activate distinct signaling pathways to drive regenerative proliferation and whether both types of tissue disruption target the same proliferative program is less well understood.

Many tissues that undergo regenerative proliferation increase the number of proliferating cells. For example, quiescent stem cells which reenter the cell cycle can support tissue repair [13, 14]. Yet other models increase cell numbers by stimulating cell cycle acceleration [15, 16]. Importantly, both strategies may co-exist. However, each must be controlled by distinct mechanisms. Cell cycle re-entry necessitates control at the G1/S transition, whereas cell cycle acceleration must control progression through G1, S, G2 and M-phases individually [17–20]. Cell cycle acceleration has been attributed to mitogenic signals driving gap phase dynamics thereby also allowing more frequent S-phase entry [21, 22]. Acceleration of S-phases or M-phases themselves have rarely been described in regeneration [23, 24]. While S-phase length is emerging as a novel regulator of cell fate decisions [25–28], acceleration of DNA replication must be tightly controlled to prevent replication stress. Not surprisingly, replication stress can drive diseases, such as cancer [29–32].

To better understand how the type of tissue damage, proliferative signals and cell cycle controls are integrated during regeneration, we chose to investigate compensatory proliferation in *Drosophila* imaginal discs [33–35]. In imaginal discs, compensatory proliferation is mediated by resident cells near the site of damage [15, 33, 34, 36] and is regulated by the conserved TNFα/JNK/AP-1, Cytokine/JAK/STAT, EGF/ERK, Myc and Hippo/Yki signaling pathways [11, 37–39]. However, like in other regeneration models, little is known about how these signals may be adapted to different types of epithelial damage and if these very different signaling pathways converge on the same cell cycle program to drive compensatory proliferation. Previous studies explored cell cycle alteration during regenerative proliferation in imaginal discs [40], but the unexpected complexity of spatial organization of signaling and cell cycle patterns in damaged discs [35, 41] invited a renewed analysis of this question.

## Results

### Compensatory proliferation in *hid-*expressing discs is associated with short G1, G2 and S-phases

**Fig 1:**
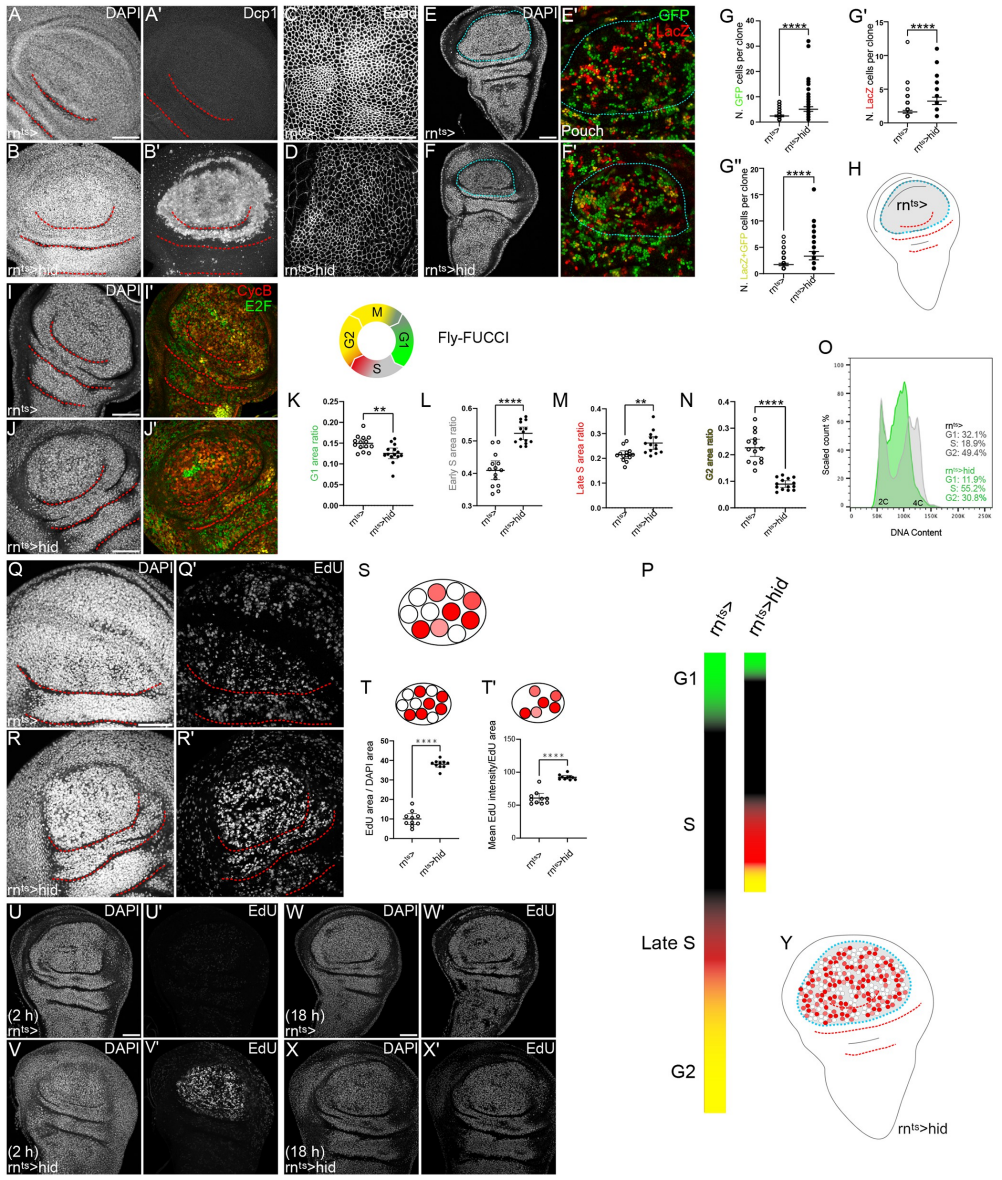
Compensatory proliferation in *hid-*expressing discs is associated with short G1, G2 and S-phases. **(A,B)** Control wing disc (A) and wing disc after 24 h of *hid-*expression in the pouch domain (B). Discs were stained with DAPI to visualize nuclei (A,B) and cleaved Dcp1 (A’,B’), a marker of apoptosis. **(C,D)** Control wing disc (C) and wing disc after 24 h of *hid-*expression in the pouch domain (D). Discs were stained for E-Cadherin to label adherens junctions. **(E,F)** Control wing disc (E,E’) and wing disc after 18 h of *hid-*expression in the pouch domain and 6 h into the recovery period (F,F’). Discs express two ‘flip-out’ construct to generate labelled clones, either controlling expression of GFP (green) or of Lac-Z (red). As both constructs are induced independently, clones either express GFP (green), LacZ (red) or both (yellow). Magnified views of pouch domain shown in (E’,F’). **(G)** Quantification of number of cells per clone expressing either GFP, LacZ or both in the pouch domain. Mean and 95% confidence interval (CI) are shown, Welch’s test was performed to test for statistical significance. (G) GFP clones (green), WT, n = 84 clones and Hid, n = 70 clones, **** <0.0001. (G’) Lac-Z clones (red), WT, n = 83 clones and Hid, n = 50 clones, **p = 0.0038. (G’) GFP and Lac-Z clones (yellow), WT, n = 75 clones and Hid, n = 43 clones, **** <0.0001. **(H)** Schematic representation of the imaginal wing disc. The *rotund*-GAL4 expressing domain is indicated in grey and a blue dotted line. Characteristic folds in the pouch, hinge and notum are represented by red dotted lines. **(I,J)** Control wing disc (I) and wing disc after 24 h of *hid-*expression in the pouch domain (J). Discs were stained with DAPI to visualize nuclei (I, J) and express the FUCCI reporter system of *ubi*-GFP-E2f1^1-230^ (green in overlay) and *ubi*-mRFP-NLS-CycB^1-266^ (red in overlay). Cells in G1 express GFP, cells in early S-phase lack expression of both FUCCI constructs, cells in late S-phase express RFP, cells in G2 express both reporters. See also S1H–S1J Fig for characterization. **(K-N)** Quantification of FUCCI profiles to determine cell cycle phase distribution for each genotype. G1 (K), Early S (L), Late S (M), G2 (N). n = 14 discs for each genotype, Welch’s test was performed to test for statistical significance. Quantifications were performed in lateral sections, thereby omitting apical mitotic cells (M-phase) from the analysis. **(O)** Flow cytometry analysis of DNA content in the pouch of undamaged control wing discs (grey) and wing disc after 24 h of *hid-*expression (green). The pouch of the wing disc was labeled by *rnGAL4*-driven expression of *UAS-GFP* and only GFP-positive flow cytometry events were plotted as counts scaled to mode against fluorescence intensity of the DNA stain Hoechst. GFP-negative events outside the pouch domain are plotted in S1K Fig. **(P)** Schematic representation of relative cell cycle length and cell cycle phase distribution in undamaged control tissues and in tissues undergoing compensatory proliferation after 24 h of *hid-*expression. **(Q,R)** Control wing disc (Q) and wing disc after 24 h of *hid-*expression in the pouch domain (R) were assessed for DNA replication activity by EdU incorporation. **(S)** Schematic representation of wing disc tissue; white nuclei do not incorporate EdU, red nuclei incorporate EdU, different shades of red visualize intensity of EdU incorporation. **(T)** Quantification of the percentage of DAPI area in the pouch domain of the wing disc that are positive for EdU incorporation. This serves as a proxy for the number of nuclei undergoing DNA replication. Mean and 95% CI are shown. Welch’s test was performed to test for statistical significance (WT, n = 10 discs, Hid, n = 10 discs, ****p = <0.0001). **(T’)** Quantification of incorporated EdU intensity in the pouch of the wing disc, measured as the mean EdU intensity within the EdU area of the pouch. This serves as a proxy for the speed of nucleotide incorporation during S-phase. Mean and 95% CI are shown. Welch’s test was performed to test for statistical significance (WT, n = 10 discs, Hid, n = 10 discs, ****p = <0.0001). **(U-X)** Control wing disc (U,W) and wing disc after 24 h of *hid-*expression in the pouch domain (V,X) were assessed for DNA replication activity by allowing larvae to feed on EdU for 2 h (U,V) and 18 h (W,X) into the recovery period. Imaging conditions were adjusted for each timepoint individually. **(Y)** Schematic representation of wing disc tissue visualizing localization of compensatory proliferation. Graphs display mean and 95% confidence interval (CI). Maximum projections of multiple confocal sections are shown in (A,B,I,J,Q,R,U,V,W,X); single sections are shown in (E,F); Local Z Projector was used to generate (C,D). Scale bars: 50 μm. Dotted lines (red) outline stereotypic folds in the wing discs.

To understand how the cell cycle may be adapted during regeneration, we examined two very distinct models of wing disc damage [33–35]. Briefly, we induced apoptosis in the wing pouch by expression of the pro-apoptotic transgenes *head involution defective (hid)* or *eiger (egr)* under the control of *rn(rotund)-*GAL4 and a temperature-sensitive GAL80^ts^ for 24 h during third instar stages (see Fig 1H). Both cell ablation systems have been previously demonstrated to undergo compensatory proliferation to regenerate the damaged disc [33–35]. Importantly, regenerative responses can be detected as early as 7–8 h after *hid* or *egr-*expression is initiated (S1A–S1D Fig) and continue well into subsequent recovery periods [41, 42]. An analysis of the imaginal discs directly after 24 h of *hid* or *egr-*expression therefore captures both the characteristics of the tissue damage, as well as the immediate regenerative responses.

In *hid-*expressing disc, the IAP-inhibitor Hid induces cell death by directly activating caspases [43] and results in controlled delamination of apoptotic cells (Fig 1A and 1B). While this causes a reduction in cell density and tissue size, this process maintains junctional integrity, epithelial barrier and planar disc morphology intact (Fig 1C and 1D). Consequently, only low activity of the central epithelial stress response pathway JNK are observed in *hid-*expressing disc, which is normally robustly activated by loss of epithelial polarity and integrity (Fig 3A and 3B) [6]. Thus, *hid-*driven cell ablation transiently challenges epithelial homeostasis by elevating the rate of cell death, and therefore models an environment of non-inflammatory, accelerated tissue turn*-*over where epithelial barrier function is maintained.

Previous studies demonstrated that a local increase in mitotic divisions drives compensatory proliferation in *hid-*expressing discs. Specifically, clones within the pouch and proximal hinge grow to larger sizes than in the disc periphery [34]. We confirmed these observations by comparing the size of TIE-DYE clones marked independently by expression of LacZ or GFP in control and *hid-*expressing discs (Fig 1E–1G) [44]. Control clones in the pouch usually contained 2.3 cells indicating that they had divided 1.2 times since clone induction 24 h prior, placing the length of one cell cycle at 20.2 h. Clones in the *hid-*expressing pouch contained on average 4.9 cells, indicating that they had divided 2.3 times in the same span of time, placing the length of one cell cycle at 10.6 h. The real cell cycle length in *hid-*expressing discs is likely shorter than this estimate, as cell survival and clone growth is limited by *hid-*induced apoptosis, which occur at the same time as clone growth. Of note, clone sizes in more peripheral tissues, such as the notum, are comparable between control and ablated discs, demonstrating that compensatory proliferation in *hid-*expressing disc is regulated by local signals at the site of *hid-*induced cell death (S1E–S1G Fig).

To better characterize the cellular program of regenerative proliferation, we analyzed the cell cycle in *hid-*expressing discs. We utilized the FUCCI cell cycle reporter, EdU incorporation assays and flow cytometry to specifically describe G1, G2 and S-phase dynamics. The FUCCI cell cycle reporter expresses GFP- and RFP-tagged peptides of the cell cycle genes E2f1 and Cyclin B, which are degraded in a cell cycle-dependent manner [45]. Correlating GFP and RFP levels with EdU incorporation patterns allowed us to establish a precise FUCCI read-out for G1, early S-phase, late S-phase and G2 cells in our hands (S1H–S1J Fig). We then asked if the cell cycle changed during compensatory proliferation and analyzed basolateral tissue sections, which omitted apically localize M-phase cells from the assay (see also S1I Fig). Of note, M-phase is a relatively low frequency event, which reflects the short time cells spend in mitosis, and thus not central to our analysis (see [42] and also S2A Fig). Strikingly, cells in the pouch domain of *hid-*expressing discs displayed a gap-phase profile different from control discs (Fig 1I and 1J). The proportion of cells in G1 and G2 was significantly reduced. In contrast, the proportion of cells in early and late S-phase was strongly increased (Fig 1K–1N). Indeed, flow cytometry of UAS-GFP-labelled cells from the *rn-GAL4*, *hid-*expressing domain confirmed a dramatic shift towards a S-phase dominated cell cycle profile, which was absent in cells outside the *rn-*GAL4 domain (Figs 1O and S1K). These observations are further supported by high nuclear area fractions of EdU incorporation in the pouch domain, indicative of more DNA-replicating cells (Fig 1Q–1T). Combining the clone growth and cell cycle analysis in *hid-*expressing discs reveals that a very short cell cycle drives compensatory proliferation when the wing disc is challenged by massive cell death. While our conclusion on reduced length of the cell cycle is in agreement with previous reports [15, 33, 34, 46], we specifically demonstrate that the cell cycle is characterized by dramatic gap phase shortening. Moreover, even though the proportion of cells in early and late S-phase is high in regenerating discs, the overall length of S-phase must also be shortened to match the extent of cell cycle acceleration (Fig 1P).

Our finding that S-phase is shortened in compensatory proliferation was unexpected. A shortened S-phase would require accelerated DNA replication to replicate the genome. Indeed, compensatory proliferation in *hid-*expressing discs was not just characterized by a high nuclear area fraction of EdU incorporation, reflecting more DNA-replicating cells. In replicating cells, EdU intensities were strongly elevated, indicating that EdU was incorporated at higher-than-normal rates into the DNA of these cells (Fig 1T’). Importantly, high rates of EdU incorporation were not caused by endoreplication, an alternative regenerative strategy reported for other tissues *in vivo* (S1L Fig) [47–50]. Elevated EdU incorporation was also not an artifact of locally altered EdU uptake due to disturbed epithelial barrier function. Neither disruption of the epithelial barrier by knock-down of the septate junction protein Cora, nor disruption of the basement membrane by targeted expression of MMP1 or MMP2, altered nucleotide incorporation in control discs (S1M–S1Q Fig). To provide further evidence that DNA replication and thus S-phase is accelerated and that endoreplication does not occur, we tested if EdU incorporation in control and *hid-*expressing tissues is ultimately reaching the same saturation levels after one round of DNA replication. We thus fed EdU to larvae for 2 h or 18 h during regeneration. After 2 h of EdU incorporation *in vivo*, we observed the expected differences in both area and intensity of EdU incorporation between control and *hid-*expressing discs, confirming that cells enter S-phase frequently and undergo accelerated DNA replication in *hid-*expressing discs (Fig 1U and 1V). However, after 18 h of EdU incorporation, EdU was incorporated equally across control and *hid-*expressing wing discs suggesting that cells had gone through S-phase at least once and that EdU incorporation was saturated at comparable levels (Fig 1W and 1X). Combined, our data demonstrate that surviving cells inside the *hid-*expressing domain undergo compensatory proliferation, and that the short compensatory cell cycle is characterized by short gap phases, and importantly, by a short S-phase facilitated by accelerated DNA replication (Fig 1P and 1Y).

### Non-autonomous proliferation in *egr-*expressing discs is also associated with S-phase acceleration

**Fig 2:**
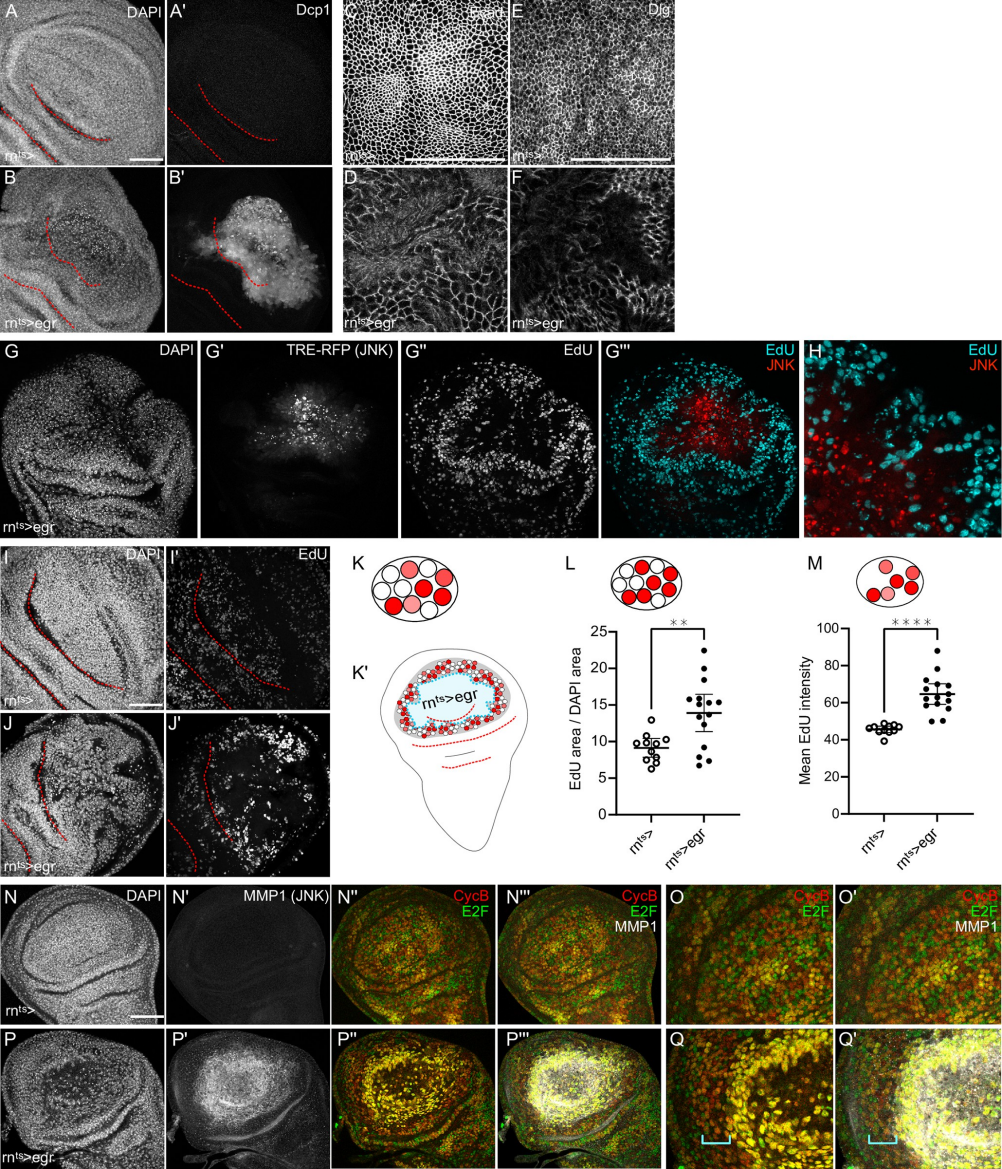
Non-autonomous proliferation in *egr-*expressing discs is also associated with S-phase acceleration. **(A,B)** Control wing disc (A) and wing disc after 24 h of *egr-*expression in the pouch domain (B). Discs were stained with DAPI to visualize nuclei (A,B) and cleaved Dcp1 (A’,B’), a marker of apoptosis. Control wing disc also shown in Fig 1A and 1A’. **(C-F)** Control wing disc (C,E) and wing disc after 24 h of *egr-*expression in the pouch domain (D,F). Discs were stained for E-Cadherin (Ecad) to label adherens junctions (C,D) and for Discs-large (Dlg) to asses apical-basal polarity (E,F). **(G,H)** Wing disc after 24 h of *egr-*expression in the pouch domain stained with DAPI to visualize nuclei (G). Discs also express the JNK activity reporter TRE-RFP (G’, red in G”‘ and H) and were assessed for DNA replication activity by EdU incorporation (G”, cyan in G”‘,H). Magnified section shown in (H). **(I,J)** Control wing disc (I) and wing disc after 24 h of *egr-*expression in the pouch domain (J). Discs were assessed for DNA replication activity by EdU incorporation (I’,J’). **(K)** Schematic representation of nuclei in wing disc tissue. White nuclei do not incorporate EdU, red-shaded nuclei incorporate EdU. Different shades represent intensity of detected EdU. **(K’)** Schematic representation of localization of compensatory proliferation in *egr-*expressing wing discs. **(L)** Quantification of the percentage of cells in the pouch domain of the wing disc that are positive for EdU incorporation, mean and 95% CI are shown. Welch’s test was performed to test for statistical significance. (WT, n = 10 discs, egr, n = 10 discs, **p = <0.0083) **(M)** Quantification of incorporated EdU intensity in the pouch of the wing disc, mean and 95% CI are shown. Welch’s test was performed to test for statistical significance (WT, n = 10 discs, Eig, n = 10 discs, ****p = <0.0001). **(N-Q)** Control wing disc (N,O) and wing disc after 24 h of *egr-*expression in the pouch domain (P,Q). Discs were stained with DAPI to visualize nuclei (N,P) for MMP-1 (a JNK target gene) to visualize JNK activity, (N‘,P‘) and express the FUCCI reporter system of *ubi*-GFP-E2f1^1-230^ (green in overlay N”-O‘) and *ubi*-mRFP-NLS-CycB^1-266^ (red in overlay, P”-Q‘). Cells in G1 express GFP, cells in early S-phase lack expression of both FUCCI constructs, cells in late S-phase express RFP, cells in G2 express both reporters. See also S1H–S1J Fig for characterization. Maximum projections of multiple confocal sections are shown in (A,B,C,G); Local Z Projector was used to generate (D,E,F); single sections are shown in (K,L,N,O,P,Q). Scale bars: 50 μm. Dotted lines (red) outline stereotypic folds in the wing discs.

**Fig 3:**
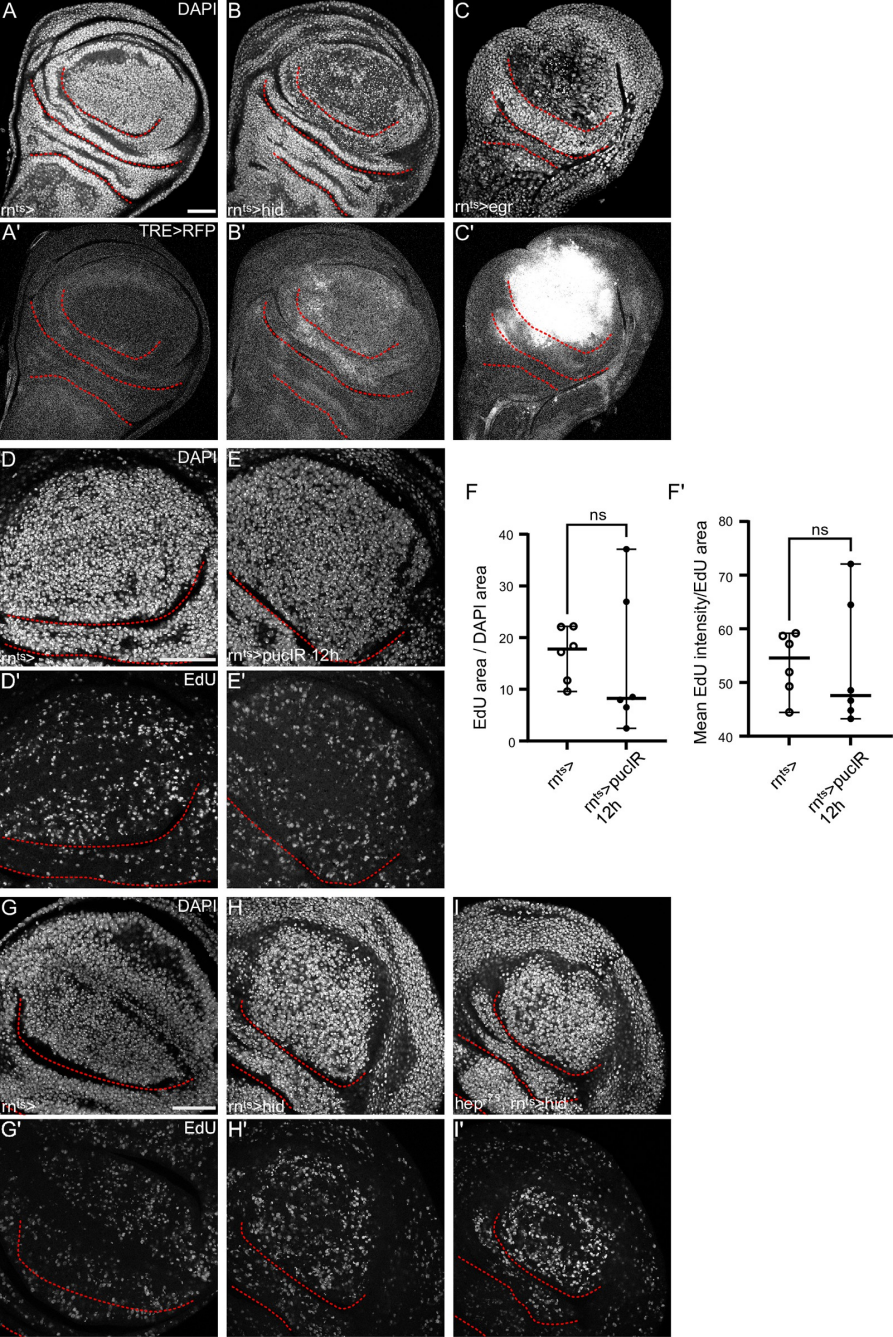
JNK signaling cannot cell-autonomously promote cell cycle acceleration. **(A-C)** Control wing disc (A) and wing disc after 24 h of *hid-*expression (B) and after 24 h of *egr-*expression (C) in the pouch domain. Discs were stained with DAPI to visualize nuclei (A-C). JNK activity is detected by activation of the TRE-RFP reporter (A’-C’). **(D,E)** Control wing disc (D) and wing disc after 12 h of *puc*-RNAi expression in the pouch domain (E). Discs were stained with DAPI to visualize nuclei (D,E). Discs were assessed for DNA replication activity by EdU incorporation (D’,E’). **(F)** Quantification of the percentage of DAPI area in the pouch domain of the wing disc that are positive for EdU incorporation. This serves as a proxy for the number of nuclei undergoing DNA replication. Mean and 95% CI are shown. Welch’s test was performed to test for statistical significance (WT, n = 6 discs, *puc*-RNAi 12h, n = 6 discs, p = 0.7581). **(F’)** Quantification of incorporated EdU intensity in the pouch of the wing disc, measured as the mean EdU intensity within the EdU area of the pouch. This serves as a proxy for the speed of nucleotide incorporation during S-phase. Mean and 95% CI are shown. Welch’s test was performed to test for statistical significance (WT, n = 6 discs, *puc*-RNAi 12h, n = 6 discs, p = 0.9774). **(G-I)** Control wing disc (G) and wing disc after 24 h of *hid-*expression in the pouch domain (H), or a *hid-*expressing disc hemizygous for the hypomorphic *hep*^*R75*^ allele (I). Discs were stained with DAPI to visualize nuclei (G-I). Discs were assessed for DNA replication activity by EdU incorporation (G’-I’). Single sections are shown in all figure panels. Scale bars: 50 μm. Dotted lines (red) outline stereotypic folds in the wing discs.

To understand if accelerated DNA replication was generally associated with compensatory proliferation, we also analyzed *egr-*expressing discs. In contrast to *hid-*expressing discs, expression of the TNFα-homologue Eiger strongly activates the epithelial stress response pathway JNK via receptor-mediated signaling [51]. This drives apoptosis, but also disrupts overall tissue architecture, junctional integrity and epithelial polarity (Fig 2A–2G, also compare Fig 3A–3C) [33, 35]. Due to the high activation of a JNK-dependent stress response program, *egr-*expression reproduces many hallmarks of highly inflammatory wounds [35, 41, 52]. Nevertheless, *egr-*expressing discs undergo compensatory proliferation to regenerate the damage [33–35]. Compensatory proliferation, however, straddles the high JNK-signaling domain, which is created by *rn-*GAL4-driven *egr-*expression in the pouch, and which cell-autonomously represses proliferation by inducing G2-arrested cells [35]. Thus, JNK-signaling cells lack EdU incorporation and the mitotic marker phospho-histone 3 (Figs 2G and S2A). Yet, in the pouch periphery, larger clone sizes can be detected [33]. In EdU incorporation assay, cells with high EdU area fraction and, importantly, increased EdU intensities form a ring around the JNK-signaling domain (Fig 2I–2M). Analysis of these cells by flow cytometry is hampered by the lack of genetic labeling opportunities. However, we analyzed the FUCCI-profile in the band of cells just outside the JNK-signaling domain. This analysis confirmed the presence of highly G2-arrested cells in the JNK-signaling domain (Fig 2N and 2P). Yet, just outside the G2-shifted JNK-signaling domain, a band of cells with reduced G1 and late G2-phase markers, but elevated markers for early and late S-phase could be observed (Fig 2O and 2Q). Combined, this data suggests that the compensatory cell cycle in *hid-* and *egr-*expressing discs is characterized by a short gap phases and accelerated S-phases. This is a surprising conclusion, as DNA replication speed during S-phase may need to be restrained to prevent replicative stress, whereas gap phases may be more safely exploited to accelerate cellular growth and cell cycle progression.

Lastly, levels of EdU incorporation in *hid-* and *egr-*expressing wing discs remained low in the notum, supporting the notion that cell cycle and S-phase acceleration are controlled by the local signaling environment of tissue damage (S2B–S2E Fig). However, in *egr-*expressing discs, the accelerated cell cycle is not directly localized in the domain of cell death as in *hid-*expressing discs, indicating that different local and non-autonomous cues may be involved in the regenerative process (compare Fig 1Y and Fig 2K’).

### JNK signaling cannot cell-autonomously promote cell cycle acceleration

To begin to understand how the accelerated cell cycle was regulated, we asked if our observations may reflect a reversion to a more primordial cell cycle, i.e. one used during rapid growth in earlier development. We thus analyzed EdU incorporation and FUCCI profiles in wing discs throughout larval development (D5-D8 AEL). Importantly, EdU incorporation rates in early stages were comparable to those in late developmental stages, and lower than those observed in *hid-*expressing discs (S3A–S3E Fig). Similarly, the analysis of the FUCCI profile confirmed a developmentally regulated increase of cells in G2 which was matched by a relative decrease of G1 cells (S3F–S3J Fig). Thus, the compensatory cell cycle does not reflect early developmental features, a conclusion supported by previous studies [40].

To understand which signaling pathways may then be required to produce a compensatory cell cycle profile, we closely analyzed the signaling environment in *hid-* and *egr-*expressing domain. As accelerated DNA replication clearly defines the compensatory cell cycle, we used EdU incorporation to faithfully track proliferative domains in both systems. We first mapped activity of the most central stress coordinator JNK, which was previously shown to modulate the cell cycle cell-autonomously [35]. Based on the JNK-reporter TRE>RFP, *hid-*expressing discs displayed mildly elevated TRE>RFP activity in the pouch where cells undergo compensatory proliferation (Figs 3A, 3B and S3K–S3M), whereas proliferating cell in *egr-*expressing discs localized just outside the very high JNK-signaling domain (Fig 2G). Thus, low levels of JNK can be detected in proliferating cells of both models. We thus asked if very low levels of JNK may somehow cell-autonomously support progression through a compensatory cell cycle. We therefore tested if independently activating JNK at mild levels was sufficient to promote EdU incorporation. A brief knock-down of the negative JNK regulator *puckered* [53] in wing discs caused low levels of JNK-associated cell death (S3N and S3O Fig). Yet, these discs did not exhibit elevated proliferation nor EdU incorporation (Fig 3D–3F). Conversely, we found that a *hid-*expressing wing disc hemizygous for the *hep*^*R75*^ JNKK-allele [35] did not display any changes to EdU incorporation patterns in the pouch (Fig 3G–3I). Similarly, expression of a dominant-negative JNK (bsk^DN^) [35] in wild type wing discs or in *hid-*expressing discs did not alter EdU incorporation dynamics (S3P–S3R Fig). Combined, these observations suggest that low JNK activity cannot cell-autonomously account for an accelerated cell cycle and S-phase profiles. This conclusion is consistent with the reported opposite role of JNK in promoting cell cycle stalling and even arrest in the G2-phase [35].

### Yorkie activity and ERK signaling are elevated in proliferating cells of *hid-*expressing discs

To understand which signaling pathways may then be required to produce a compensatory cell cycle profile, we closely analyzed the signaling environment in *hid-*expressing disc. We focused on pathways known to promote proliferation during tissue regeneration, specifically the growth-promoting and pro-survival pathways Hippo/Yki, Ras/ERK, JAK/STAT and Myc, predicting that the regulation of these pathways would positively correlate with high EdU intensity in *hid-* or *egr-*expressing discs.

**Fig 4:**
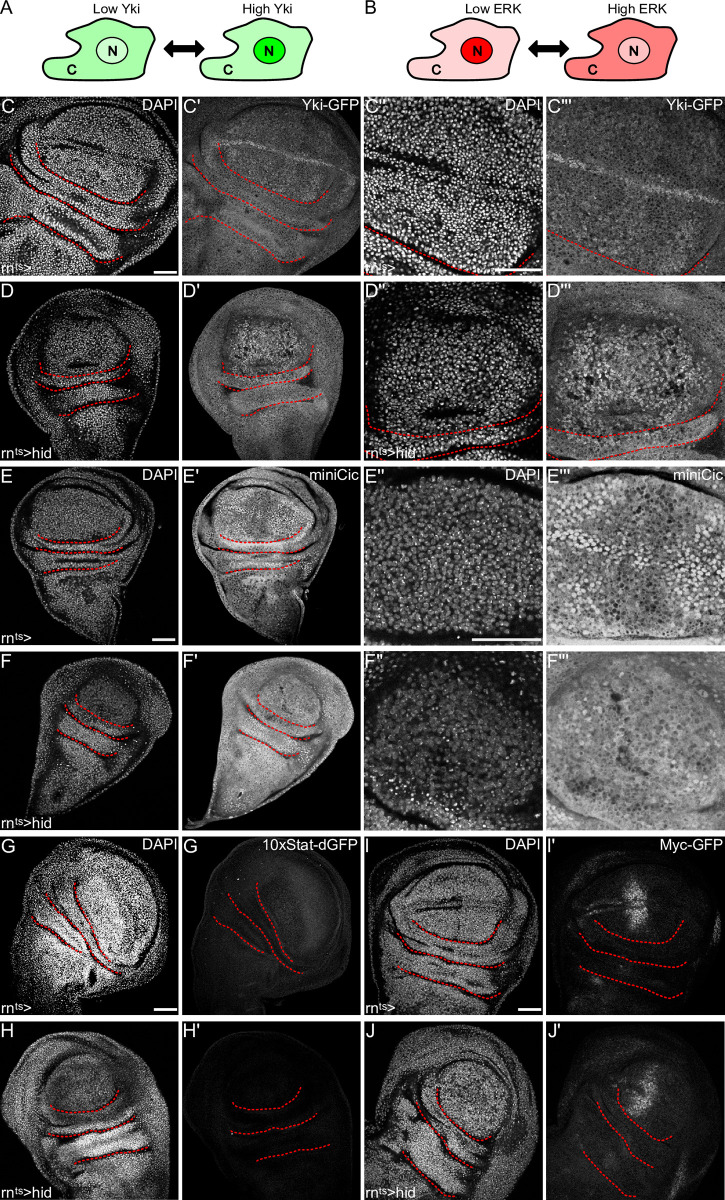
Yorkie activity and ERK signaling are elevated in proliferating cells of *hid-*expressing discs. **(A)** Schematic representation of nuclear shuttling of Yki-GFP. High levels of nuclear Yki-GFP represent Yki-activation. **(B)** Schematic representation of nuclear shuttling of the miniCic-mCherry reporter. Low levels of nuclear miniCic represent high ERK activity and vice versa. **(C-F)** Control wing disc (C,E) and wing disc after 24 h of *hid-*expression in the pouch domain (D,F). Discs either express Yorkie-GFP (C, D) or the ERK reporter miniCic-mCherry (E,F). Magnified view of the pouch domain (C”-F”). Discs were stained with DAPI to visualize nuclei. **(G-J)** Control wing disc (G,I) and wing disc after 24 h of *hid-*expression in the pouch domain (H,J). Discs either express the JAK/STAT reporter *10xStat92E>dGFP* (G,H) or an endogenously tagged Myc-GFP construct (I,J). Discs were stained with DAPI to visualize nuclei. Maximum projections of multiple confocal sections are shown in (G,H,I,J); single sections are shown in (C,D,E,F). Scale bars: 50 μm. Dotted lines (red) outline stereotypic folds in the wing discs.

We first monitored signaling through the Hippo/Yki pathway by nuclear localization of the effector Yorkie (Yki) [54]. Strikingly, Yki distinctly localized to nuclei in proliferating cells in the *hid-*expressing pouch, but not in normally cycling cells in the disc periphery (Figs 4A, 4C, 4D, S4A and S4B). Similarly, when we monitored signaling through the ERK pathway using the miniCic reporter system [55], we found that ERK signaling was specifically elevated in proliferating cells of *hid-*expressing discs (Figs 4B, 4E, 4F, S4C and S4D). Utilizing a reporter for activated STAT [56], we found that proliferating cells in *hid-*expressing discs did not activate JAK/STAT signaling (Figs 4G, 4H, S4E and S4F). Similarly, only cells of the anterior compartment maintained an ancestral Myc expression pattern also observed in undamaged control discs (Fig 4I and 4J). We conclude that Myc is not upregulated *de novo* or expressed in all proliferating cells of *hid-*expressing discs. Combined, this systematic analysis revealed that compensatory proliferation in *hid-*expressing disc highly correlates with nuclear localization of Yki and elevated ERK activity.

### JAK/STAT signaling and Myc-expression are elevated in proliferating cells of *egr-*expressing discs

**Fig 5:**
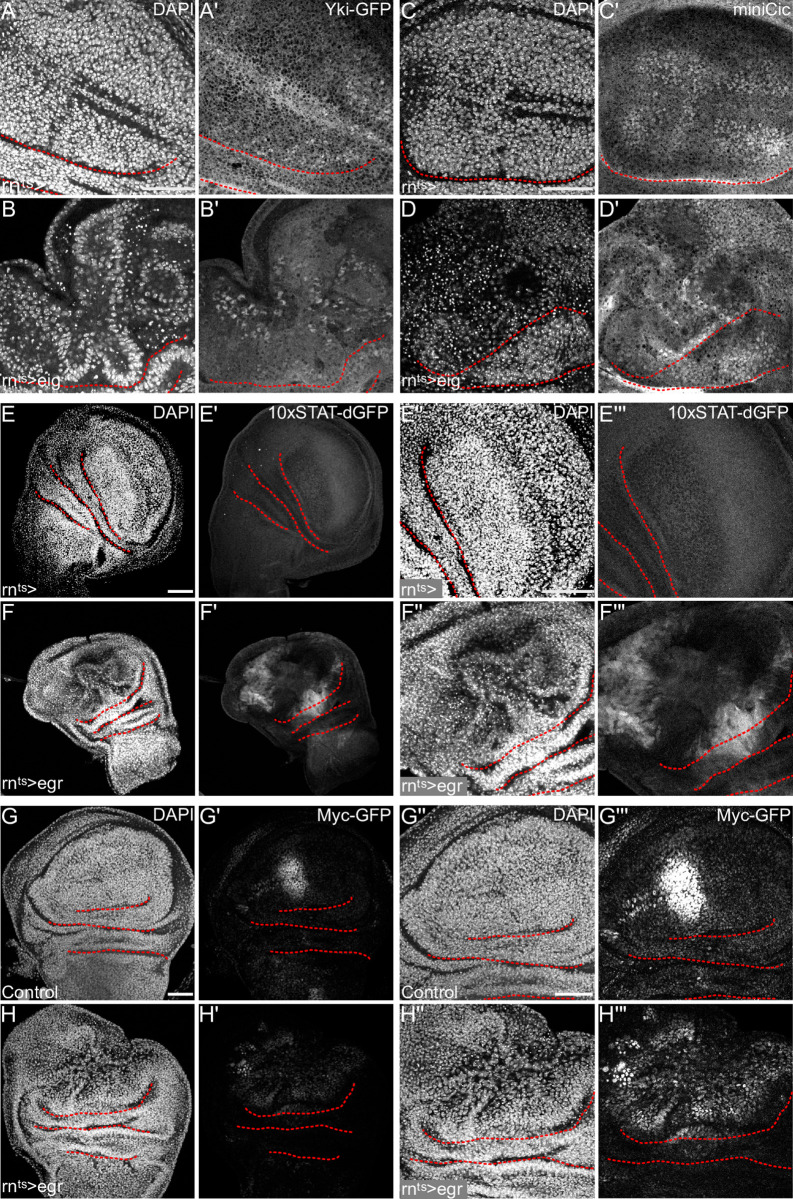
JAK/STAT signaling and Myc-expression are elevated in proliferating cells of *egr-*expressing discs. **(A-D)** Control wing disc (A,C) and wing disc after 24 h of *egr-*expression in the pouch domain (B,D). Discs either express Yorkie-GFP (A,B) or the ERK reporter miniCic-mCherry (C,D). Magnified view of the pouch domain. Discs were stained with DAPI to visualize nuclei (A-D). **(E-H)** Control wing disc (E,G) and wing disc after 24 h of *egr-*expression in the pouch domain (F,H). Discs either express the JAK/STAT reporter *10xStat92E>dGFP* (E,F) or an endogenously tagged Myc-GFP construct (G,H). Discs were stained with DAPI to visualize nuclei (E-H). Magnified view of the pouch domain (E”-H”). Images with increased brightness show the presence of Myc-GFP in the regenerative domain (G”‘,H”‘). We suggest that the Myc-expressing cells in the anterior pouch domain of control disc are killed by *egr-*expression and a new expression pattern of Myc is set up *de novo* by tissue damage signals. Maximum projections of multiple confocal sections are shown in (E,F,G,H); single sections are shown in (A-D). Scale bars: 50 μm. Dotted lines (red) outline stereotypic folds in the wing discs.

To understand if a similar signaling signature was associated with non-autonomous compensatory proliferation in *egr-*expressing discs, we analyzed the same reporter panel for changes in the domain of proliferating cells. Strikingly, in contrast to *hid-*expressing discs, Yki was not enriched in nuclei of proliferating cells but instead localized to the nuclei of cell cycle arrested, high JNK-signaling cells in *egr-*expressing discs, as reported before (Figs 5A, 5B, S5A and S5B) [57]. Similarly, no consistent correlation could be detected for ERK-activation in proliferating cells of *egr-*expressing discs (Figs 5C, 5D, S5C and S5D). However, in contrast to *hid-*expressing discs, domains of compensatory proliferation correlated well with activation of the JAK/STAT reporter (Figs 5E, 5F, S5E and S5F) and *de novo* expression of Myc in the peripheral pouch and hinge domains, a region where it is normally not expressed (Fig 5G and 5H). Importantly, the pattern of this signaling signature did not change during regeneration and could still be detected 24 h after *egr-*expression well into the recovery period (S5G–S5N Fig). Of note, hid-expressing discs maintained their signaling signature as well (S5O and S5P Fig). Combined, we find that JAK/STAT activity and Myc expression are specifically detected in cells undergoing compensatory proliferation that must be driven by non-autonomous signaling from *egr-*expressing domains. Indeed, JAK/STAT-activating, secreted ligands of the Unpaired family are expressed in JNK-signaling cells [41, 42, 52, 58–60].

As a result, this analysis left us with the surprising conclusion, that completely different signaling signatures can be associated with compensatory proliferation and specifically, with accelerated nucleotide incorporation and thus DNA replication speed. These results suggest that at least two distinct regulatory circuits may converged on compensatory proliferation and the same cell cycle adaptation upon distinct damaging challenges.

### Yki and EGF cooperate to drive compensatory proliferation in response to non-inflammatory damage

**Fig 6:**
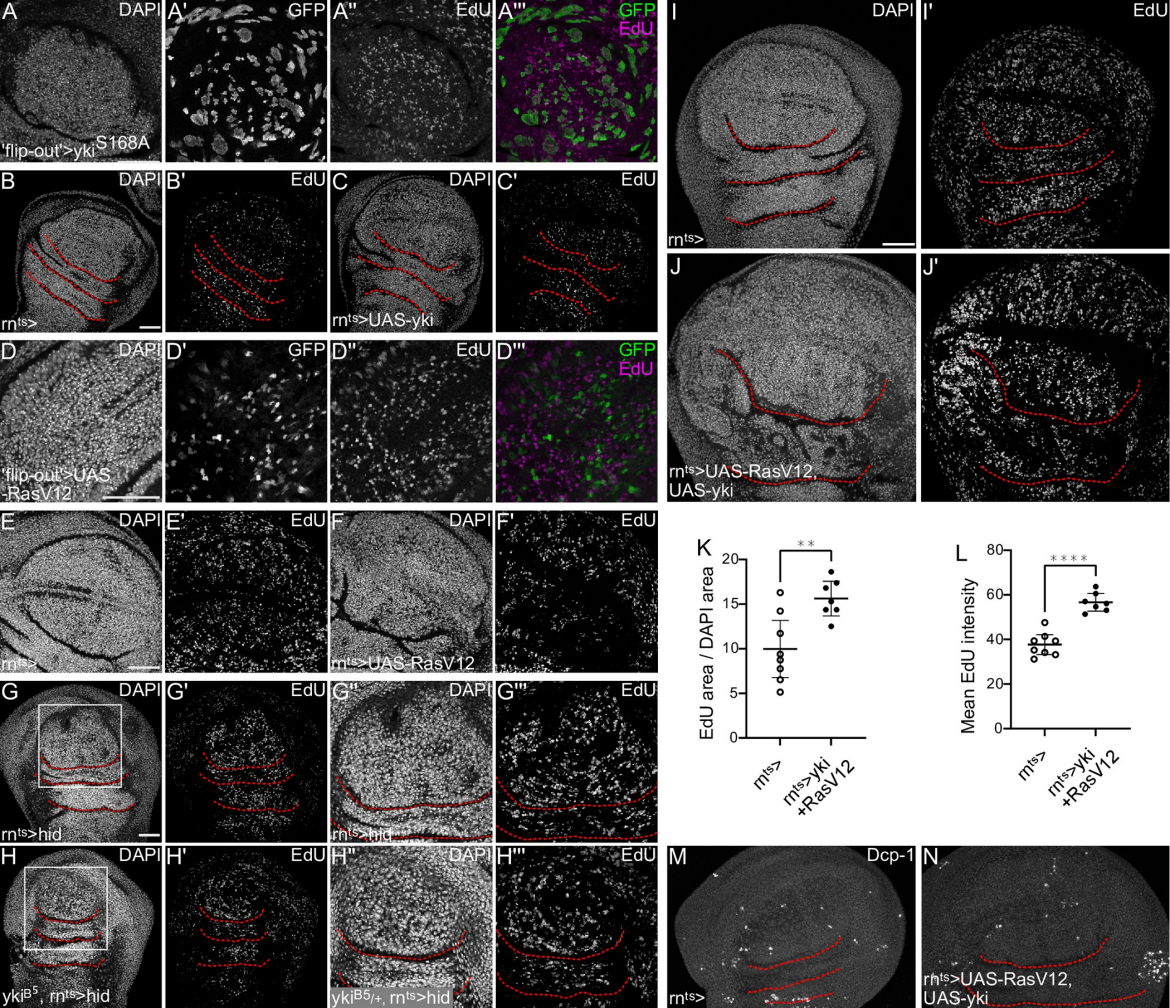
Yki and ERK cooperate to drive compensatory proliferation in response to non-inflammatory damage. **(A)** A wing disc expressing the act-GAL4 ‘flip-out’ system controlling the mosaic expression of GFP and UAS-yki.S168A (green in A”‘). Discs were stained with DAPI to visualize nuclei and were assessed for DNA replication activity by EdU incorporation (magenta). **(B, C)** Control wing disc (B), wing disc after 24 h of UAS-yki expression in the pouch domain (C). Discs were stained with DAPI to visualize nuclei and were assessed for DNA replication activity by EdU incorporation. **(D)** A wing disc expressing the act-GAL4 ‘flip-out’ system controlling the mosaic expression of GFP and UAS-Ras^V12^ (green in D”‘). Discs were stained with DAPI to visualize nuclei and were assessed for DNA replication activity by EdU incorporation (magenta). **(E,F)** Control wing disc (E) and wing disc after 24 h of UAS-Ras^V12^ expression in the pouch domain (F). Discs were stained with DAPI to visualize nuclei and were assessed for DNA replication activity by EdU incorporation. **(G,H)** Wing disc after 24 h of *hid-*expression (G) and a wing disc heterozygous for *yki* ^*B5*^ after 24 h of *hid-*expression (H). Discs were stained with DAPI to visualize nuclei and were assessed for DNA replication activity by EdU incorporation. White frame marks the magnified view of the pouch domain shown in (G”-H”‘). **(I,J)** Control wing disc (I) and a wing disc after 24 h of UAS-yki-GFP and UAS-Ras^V12^ expression in the pouch domain (J). Discs were stained with DAPI to visualize nuclei and were assessed for DNA replication activity by EdU incorporation. **(K)** Quantification of the percentage of DAPI area in the pouch domain of the wing disc that was positive for incorporated EdU in control wing discs, or Yki and Ras^V12^ expressing wing discs. Mean and 95% CI are shown. Welch’s test was performed to test for statistical significance. (WT, n = 8 discs, UAS-yki-GFP, UAS-Ras^V12^, n = 7 discs, **p<0.01). **(L)** Quantification of incorporated EdU intensity, measured as the mean EdU intensity within the EdU area of the pouch in control wing discs and wing disc after 24 h of Yki and Ras^V12^ expression. A Welch’s test was performed to test for statistical significance. (WT, n = 8 discs, UAS-yki-GFP, UAS-Ras^V12^, n = 7 discs, ****p = <0.0001). **(M,N)** Control wing disc (M) and a wing disc after 24 h of UAS-yki-GFP and UAS-Ras^V12^ expression in the pouch domain (N). Discs were stained for cleaved Dcp1, a marker of apoptosis. Graphs display mean and 95% CI. Maximum projections of multiple confocal sections are shown in (G,H,I,J). Single sections are shown in (A-F). Scale bars: 50 μm. Dotted lines (red) outline stereotypic folds in the wing discs.

To investigate which of these signaling pathways may truly be required for compensatory proliferation, we systematically analyzed sufficiency and necessity of Hippo/Yki and Ras/ERK signaling in *hid-*expressing disc. We first asked if Hippo/Yki or Ras/ERK activation alone were sufficient to induce accelerated EdU incorporation. However, neither expression of a phospho-ablative Yki^S168A^ construct nor RNAi-mediated knock-down of Warts altered the rate of EdU incorporation in mosaic clones, or upon expression in the pouch (Figs 6A–6C, S6A and S6B). Similarly, expression of oncogenic Ras^V12^ alone failed to phenocopy an accelerated S-phase profile (Fig 6D–6F). However, to understand if Hippo/Yki and Ras/ERK are necessary for S-phase acceleration, we created *hid-*expressing discs heterozygous mutant for a null allele of *yki*^*B5*^. Indeed, in the very rare discs that we were able to recover due to high lethality, we observed a reduction in EdU incorporation, if compared to control discs (Figs 6G, 6H, S6C and S6D). This suggests, that Hippo/Yki is necessary to drive nucleotide incorporation during S-phase in *hid-*expressing discs. We performed experiments to test the necessity of Ras/ERK signaling in S-phase acceleration. We analyzed discs that either co-expressed a dominant-negative Egfr (Egfr^DN^) in *hid-*expressing cells (S6E–S6H Fig) or that were heterozygous for *Ras*^*1*^ (S6I–S6N Fig). Both strategies failed to reveal changes to EdU incorporation dynamics. However, it is possible that Egfr^DN^-expressing cells die too quickly in the context of *hid-*coexpression, and that *Ras*^*1*^ heterozygosity may not sufficiently interfere with ERK function. Thus, other genetic strategies may be needed to perform these experiments.

Importantly, though, as neither pathway was sufficient to accelerate nucleotide incorporation individually, we tested if Hippo/Yki and Ras/ERK pathways cooperate. Indeed, the combined expression of Ras^V12^ and Yki was sufficient to drive elevated nucleotide incorporation in the pouch (Fig 6I–6L). Not surprisingly, the pouch overgrew, demonstrating that both pathways cooperate in promoting proliferation. This was not associated with elevated levels of apoptosis, indicating that accelerate nucleotide incorporation was directly caused by Ras^V12^ and Yki co-expression (Fig 6M and 6N). Combined these observations demonstrate, that the *hid-*expressing model of local, non-inflammatory regeneration uses Hippo/Yki and Ras/ERK activation to promote cell cycle adaptations for compensatory proliferation.

### JAK/STAT and Myc are sufficient to drive S-phase acceleration in response to inflammatory damage

**Fig 7:**
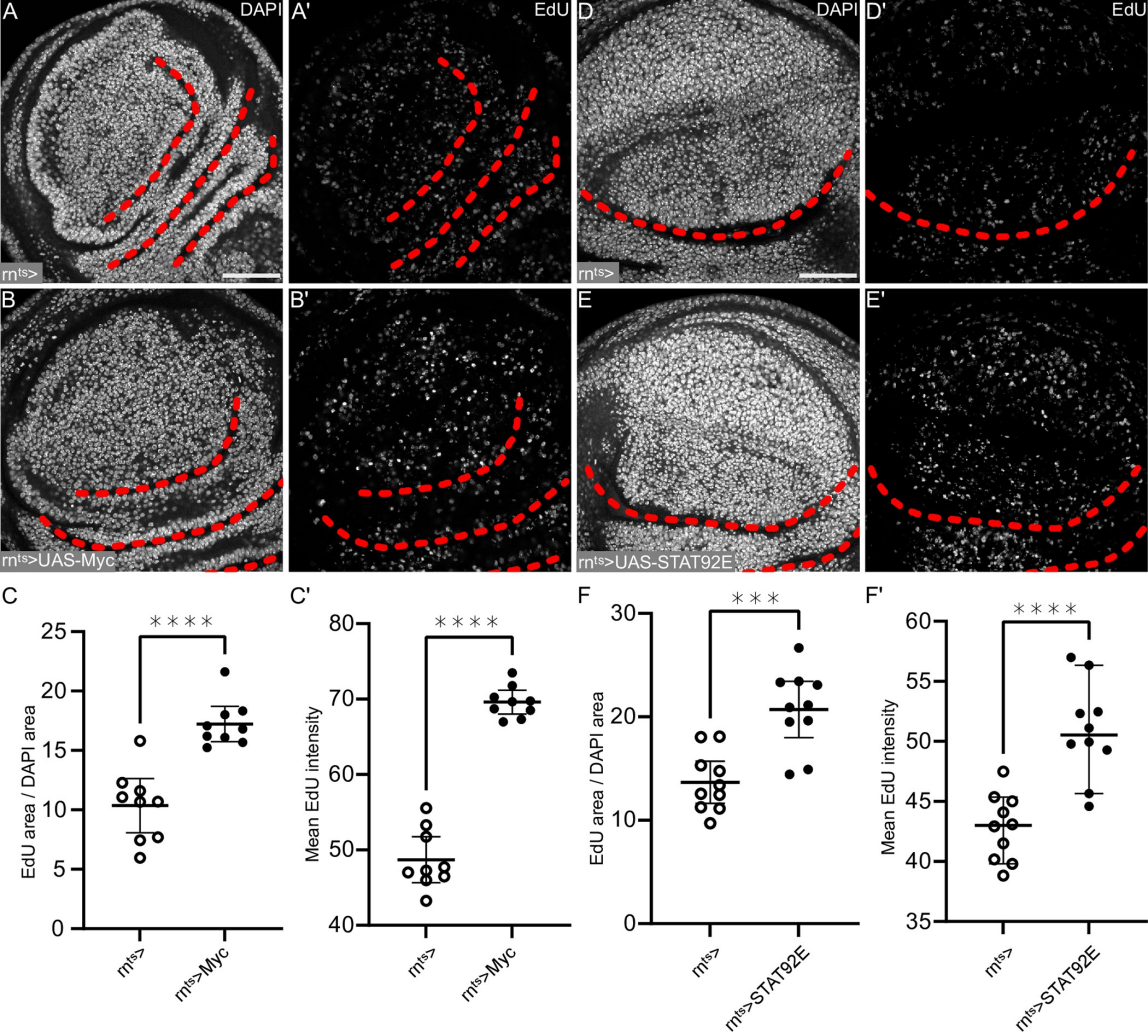
JAK/STAT and Myc are sufficient to drive S-phase acceleration in response to inflammatory damage. **(A,B)** Control wing disc (A), wing disc after 24 h of UAS-Myc expression in the pouch domain (B). Discs were stained with DAPI to visualize nuclei and were assessed for DNA replication activity by EdU incorporation. **(C)** Quantification of the percentage of DAPI areas that were positive for incorporated EdU in control wing discs or UAS-Myc expressing wing discs. This serves as a proxy for the number of nuclei undergoing DNA replication. **(C’)** Quantification of incorporated EdU, measured as mean EdU intensity in the DAPI area within the pouch. A Welch’s test was performed to test for statistical significance. (WT, n = 9 discs, UAS-Myc, n = 9 discs, ****p = <0.0001). **(D,E)** Control wing disc (D), and a wing disc after 24 h of UAS-Stat92E-expression (E). Discs were stained with DAPI to visualize nuclei and were assessed for DNA replication activity by EdU incorporation. **(F)** Quantification of the percentage of DAPI areas that were positive for incorporated EdU in control wing discs or UAS- Stat92E expressing wing discs. This serves as a proxy for the number of nuclei undergoing DNA replication. **(F’)** Quantification of incorporated EdU, measured as mean EdU intensity in the DAPI area within the pouch. Welch’s test was performed to test for statistical significance. (WT, n = 10 discs, UAS-Stat, n = 10 discs, ***p<0.001, ****p<0.0001). Graphs display mean and 95% CI. Single sections are shown in (A,B,D,E). Scale bars: 50 μm. Dotted lines (red) outline stereotypic folds in the wing discs.

Since Hippo/Yki and Ras/ERK signaling did not robustly correlate with domains of compensatory proliferation in *egr-*expressing disc, we asked if JAK/STAT activation and Myc expression may directly control cell cycle acceleration. We first tested if Myc and JAK/STAT alone were sufficient to induce accelerated EdU incorporation. Indeed, overexpression of Myc alone was sufficient to drive S-phase acceleration, aligning with mammalian reports that Myc can accelerate S-phase progression (Fig 7A–7C) [31]. Similarly, expression of the transcription factor Stat92E was sufficient to cell-autonomously drive high levels of EdU incorporation, confirming that JAK/STAT is a mitogenic pathway strongly implicated in compensatory proliferation (Fig 7D–7F) [39, 61, 62]. We wanted to understand, if Myc or Stat92E activity are rate-limiting for EdU incorporation. We thus generated *egr-*expressing discs heterozygous for the null allele *Stat92E*^*85C3*^. However, we failed to detect any changes in cells undergoing compensatory proliferation, suggesting that heterozygosity for Stat92E is not rate-limiting for EdU incorporation, or alternatively, that Myc upregulation can compensate for reduced *Stat92E* function (S7A and S7B Fig). Due to lethality of *egr-* and *hid-*expressing larvae heterozygous for *dMyc* alleles, we were unable to specifically test the necessity of Myc in mediating cell cycle acceleration. However, our observations suggest that activation of JAK/STAT signaling or elevated expression of Myc alone are sufficient to accelerate DNA replication during compensatory proliferation.

### Compensatory proliferation is not associated with replication stress

**Fig 8:**
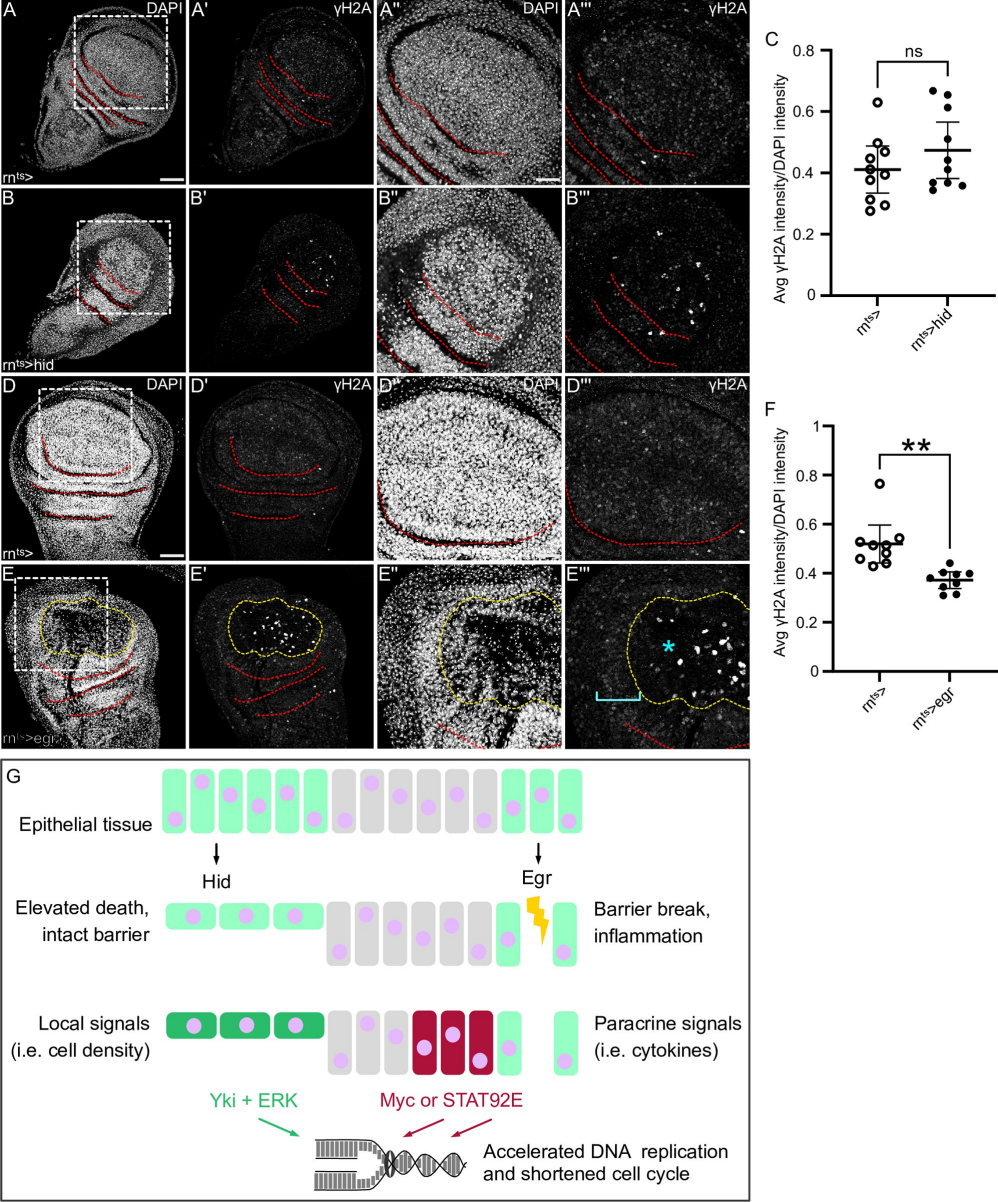
Compensatory proliferation is not associated with replication stress. **(A-C)** Control wing disc (A), and a wing disc after 24 h of *hid-*expression in the pouch domain (B). Discs were stained with DAPI to visualize nuclei and were assessed for DNA damage by staining for phosphorylated γH2A. White frame marks position of views shown in (“, “‘) panels. (C) Quantification of H2Aγ staining intensity within the DAPI area of the pouch domain normalized to DAPI intensity to correct for fluctuations in DNA density. Mean and 95% CI are shown. Welch’s test was performed to test for statistical significance. (WT, n = 10 discs, Hid, n = 10 discs, ns, p = 0.2533). **(D-F)** Control wing disc (D), and a wing disc after 24 h of *egr-*expression in the pouch domain (E). Discs were stained with DAPI to visualize nuclei and were assessed for DNA damage by staining for phosphorylated γH2A. Yellow dashed line in (E) demarcates the area of high JNK reporter activity (cyan star) as assessed by TRE-RFP expression (not shown). Compensatory proliferation occurs in a band outside of the JNK-signaling domain (cyan bracket). White frame marks position of views shown in (“, “‘) panels. (F) Quantification of H2Aγ staining intensity within the DAPI area of domain outside the JNK-signaling domain normalized to DAPI intensity to correct for fluctuations in DNA density. Welch’s test was performed to test for statistical significance (WT, n = 9 discs, Egr, n = 9 discs, **p = 0.0019). **(G)** Model of signaling environment driving compensatory proliferation and accelerated DNA replication in response to two distinct challenges to tissue health. Maximum projections of multiple confocal sections are shown in (A,B,D,E). Scale bars: 50 μm. Dotted lines (red) outline stereotypic folds in the wing discs.

Many reports highlight the emergence of replicative stress upon pathological acceleration of DNA replication, for example in tumors [29–32]. To understand if accelerated DNA replication during compensatory proliferation was associated with elevated replication stress, we assessed levels of DNA double-strand breaks in *hid-* and *egr-*expressing discs [63, 64]. While very occasionally apoptotic cells displayed high levels of phosphorylated H2Av staining, we failed to detect a general increase in this DNA damage marker in areas of compensatory proliferation (Fig 8A–8F). This suggests that mechanisms exist which ensure that accelerated DNA replication does not generally cause replication stress and DNA damage. It suggests that DNA-replication can be safely accelerated to increase cell cycle progression. Even though we report here that S-phases are accelerated, we also observe that gap phases nearly disappear, suggesting that, ultimately, safe DNA replication speed is still rate-limiting for cell cycle length.

## Discussion

In this study we find that two distinct signaling signatures can drive compensatory proliferation during tissue regeneration by shortening gap phases, and surprisingly, accelerating DNA replication during S-phase (Fig 8G). We find that, in the absence of epithelial barrier damage and inflammation, *hid-*expressing discs accelerate the cell cycle by cooperativity of Hippo/Yki and Ras/ERK signaling. Hippo/Yki and Ras/ERK signaling have been reported to respond to local changes in cell density and may specifically respond to changes in the force balance of cell-cell junctions [6, 65–69]. As the cell density in *hid-*expressing discs continuously decreases but junctions remain intact, Hippo/Yki and Ras/ERK may be ideally suited to control proliferation in this non-inflammatory environment.

Inflammatory tissue damage that disrupts the epithelial barrier is characterized by high JNK activation. High JNK signaling induces a senescent cell cycle arrest which does not support proliferation [35]. Thus, compensatory proliferation must occur distally to the wound and is guided by paracrine factors secreted from the site of damage. In fact, the JAK/STAT activators of the Unpaired cytokine-like family and the Myc activator Wg of the Wnt family are expressed at high levels in *egr-*expressing and JNK-signaling cells [33, 42]. We observe strong activation of JAK/STAT and Myc outside JNK-signaling domains and either is sufficient to drive accelerated S-phase profiles in the disc. JAK/STAT signaling has been implicated in driving S-phase entry via Cyclin E expression [70]. Myc may facilitate the metabolic drive needed for cell growth in gap phases via targeting of protein synthesis and Cyclin E [71, 72]. Strikingly, metabolic drive may be sufficient for S-phase acceleration as expression of a constitutively active Insulin receptor is sufficient to promote nucleotide incorporation in EdU assays (S8A–S8C Fig). While Myc and JAK/STAT have known access points into the cell cycle, it is not clear how they would specifically promote nucleotide incorporation and thus S-phase acceleration. However, Myc expression has also been found to increase replication speed in mammalian cells [31], suggesting that conserved mechanisms may confer S-phase acceleration.

Previous studies have analysed cell cycle changes in regenerating and transdetermining imaginal discs [15, 40]. Specifically, the dissection of the ‘blastema’ cell population and subsequent analysis by flow cytometry revealed both an increase in S-phase and G2 cells, as well as an increase in cell size [40]. Based on more detailed spatial studies from our lab, we now suggest that the G2-component and large cell sizes arise from cells undergoing a JNK-controlled G2-arrest directly at the wound sites [35]. The S-phase component arises from cell undergoing compensatory proliferation next to JNK-controlled wound site (this study). Thus, our work sheds a new light on the spatial organisation of cell cycle adaptations in regenerating tissues.

Little is known about molecular details that modulate of S-phase length. S-phase length is controlled by the number of active replication forks and their velocity. The number of active replication forks could be increased via recruitment of dormant ORCs. ORC usage is lineage specific and correlates with transcriptional and epigenetic states [25, 27, 73–77]. The signaling environment of compensatory proliferation alters transcriptional activity, and as a result may recruit dormant ORCs for S-phase acceleration. In contrast, processivity and velocity of the replication complex can be regulated by components of the cell cycle machinery [78–85]. In addition, enhancing access to DNA can promote replication complex processivity. Indeed, the DNA helicase Top3a was previously identified to be genetically required for compensatory proliferation [86]. An additional function for accelerated DNA replication, that goes beyond its role in proliferation, is not known [see also 16]. However, S-phase length has been implicated in cell fate decisions [25–28]. If S-phase acceleration may thus support reprogramming of imaginal disc cells during tissue repair would form the basis of an interesting line of future research.

## Methods

### Fly genetics

All fly stocks and experimental crosses were maintained on standard media and raised at 18 °C unless otherwise specified. For detailed genotypes, please refer to S1 Table.

### Flip-out clones

GAL4/UAS-driven ‘flip-out’ experiments utilized heat-shock-driven expression of a flipase. The respective crosses were allowed to lay eggs for 72 h at 25°C followed by a heat-shock at 37°C for 5–25 min. Larvae were dissected at wandering 3rd instar stage or as indicated (30 h or 54 h after heat-shock). To analyze the growth of clones in *hid-*expressing discs, GFP was expressed under the control of a *ubiquitin* promoter upon ’flip-out’ of an FRT cassette. After a 7 minutes heat-shock at 37°C, the cross was shifted to 30°C for 18 h to activate expression of *hid* in the *rn-*GAL4 domain. Larvae were dissected and fixed 6 h into the recovery period. Nuclei were counted for each GFP-positive clone in the pouch or notum.

### Cell ablation using GAL4/UAS/GAL80ts system to express UAS-hid or UAS-egr

To induce expression of *egr* or *hid*, experiments were carried out as described in [33, 35, 42] with few modifications. Briefly, larvae of genotype *rn-GAL4*, *tub-GAL80*^*ts*^ *(rn*^*ts*^) and carrying the desired UAS-transgenes were staged by a 6 h egg collection and raised at 18°C at the density of 50 larvae/vial. Overexpression of transgenes was induced by shifting the temperature to 30°C for 24 h at day 6 or 7 after egg deposition (AED), as indicated. Larvae were subsequently dissected for analysis (recovery time point R0) or allowed to recover at 18°C for the indicated time. All images represent R0, unless noted otherwise. Control genotypes were either *rn*^*ts*^ control crosses, or the sibling larvae (*+/TM6B*, *tubGAL80*) [33]. At least 20 discs were dissected for each genotype.

### Immunofluorescence microscopy

Wing discs from third instar larvae were dissected and fixed for 15 min at room temperature (RT) in 4% paraformaldehyde in PBS. Washing steps were performed in PBS containing 0.1% TritonX-100 (PBT). Discs were then incubated with primary antibodies in PBT, gently mixing overnight at 4°C. The following antibodies were used: rabbit anti-cleaved Dcp-1 (Cell Signaling, 9578, 1:200), mouse anti-β-Galactosidase (Promega, Z3783, 1:1000), chicken anti-GFP (Abcam, ab13970, 1:1000), rabbit anti-GFP (Invitrogen, G10362, 1:200), rabbit anti-H2Av-pS137 (Rockland, 600-401-914, 1:500), mouse anti-H3-pS10 (Abcam, ab14955, 1:2000), rat anti-HA (MAB facility at the Helmholtz Zentrum München, 3F10, 1:20), mouse anti-MMP1 (DSHB, a mix of 3A6B4, 3B8D12 and 5H7B11, each 1:30), mouse anti-RFP (Abcam, ab65856, 1:100), rat anti-RFP (MAB facility at the Helmholtz Zentrum München, 5F8, 1:20). Tissues were counterstained with DAPI (0.25 ng/μl, Sigma, D9542) or Phalloidin-Alexa Fluor 488/647 (1:100, Life Technologies) or Phalloidin-conjugated TRITC (1:400, Sigma) during incubation with cross-absorbed secondary antibodies coupled to Alexa Fluorophores (Invitrogen or Abcam) at room temperature for 2 h. Tissues were mounted using SlowFade Gold Antifade (Invitrogen, S36936). Whenever possible, experimental and control discs were processed in the same vial and mounted on the same slides to ensure absolute comparability in staining conditions between different genotypes. Genotypes were distinguished on the slide by deliberately co-expressed fluorescence markers (GFP, RFP, HA, LacZ). Of note, the signals of the following fluorescent reporters were further amplified by anti-GFP or anti-mCherry antibody staining: miniCiC-mCherry, Yki-GFP. Images were acquired using the Leica TCS SP8 Microscope (DFG Project 414136422), using sample-matched confocal settings.

### EdU Labelling

EdU incorporation was performed after crude dissection and detected using the Click-iT Plus EdU Alexa Fluor 647 Imaging Kit (Invitrogen, C10640) prior to primary antibody incubation. Briefly, larval cuticles were inverted in Schneider’s medium and incubated with EdU (10μM final concentration) at RT for 15 minutes. Cuticles were then fixed in 4% PFA/PBS for 15 minutes, washed for 30 minutes in PBT 0.5%. EdU-Click-iT labeling was performed according to manufacturer’s guidelines. Tissues were washed in PBT 0.1%, after which additional immunostainings, sample processing and imaging were carried out as described above.

### BrdU Labelling

Larval cuticles were inverted in Schneider’s medium and incubated with BrdU (10μM final concentration) at RT for 15 minutes. Cuticles were then fixed in 4% PFA/PBS for 20 minutes and washed in 0.5% PBT. Samples were then incubated in HCl at 2N concentration for 45 minutes and subsequently washed twice for 2 min in 0.1M Na_3_BO_3_ pH 8.5. After three washes in PBT 0.5%, discs were incubated with mouse anti-BrdU (BD, 555627, 1:100) in PBTN, gently mixing overnight at 4°C. Tissues were washed in PBT 0.5%, then counterstained with DAPI (0.25 ng/μl, Sigma, D9542) and secondary antibody coupled to Alexa Fluorophores (Invitrogen) at room temperature for 2 h. Tissues were washed again in PBT 0.5% and PBS before mounting. Mounting and imaging were performed as described above.

### EdU feeding experiment

After *hid-*expression using the GAL4/UAS/GAL80ts system, larvae were transferred to fly food containing EdU (100 μM final concentration). Larvae were left feeding for 2 h or 18 h. Only larvae still roaming in the food were chosen to dissect after the feeding. After dissection, larvae were fixed in 4% PFA/PBS for 15 minutes, and EdU was detected using the Click-iT Plus EdU Alexa Fluor 647 Imaging Kit (Invitrogen, C10640) as described above.

### Flow cytometry

Cell cycle analysis of wing imaginal discs by flow cytometry was performed as described [87]. Wing imaginal discs from at least 10 larvae were dissected in PBS and incubated for 2 h in PBS containing 9X Trypsin-EDTA (Sigma, T4174) and 0.5 μg/ml Hoechst 33342 (Invitrogen, H3570). Cells were analyzed with an LSRFortessa cell analyzer (BD Biosciences) or FACS Aria II cell sorter (BD Biosciences). Univariate cell cycle analysis was performed using the Watson Pragmatic algorithm in FlowJo v10 (FlowJo).

### Image analysis and quantification

#### General comments

Where possible, control and experimental samples were fixed, processed and mounted together to ensure comparable staining and imaging conditions. Positive results were verified with a minimum of n = 3 replicates. Images were processed, analyzed and quantified using tools in Fiji (ImageJ v2.0.0) [88] (see below). Great care was taken to apply consistent methods (i.e. number of projected sections, thresholding methods, processing) within experimental settings. Statistical analyses were performed in Graphpad Prism (see below). Briefly, every data set was checked for normality of distribution and homogeneity of variances by applying Shapiro’s and Bartlett’s test, respectively. The α value for each analysis was set to 0.01 (α = 0.01). A Welch’s Test was then performed on one of the replicates as indicated in the figure legends. Confidence interval (95%) is shown for each dataset to show the range of the true population’s mean, for a fairer display of the variability of the sample compared to a point estimate. Statistical tests are indicated in the figure legends.

Statistical significance is shown as: * = p val<0.05, ** = p val<0.01, *** = p val<0.001, **** = p val<0.0001. Figure panels were assembled using Affinity Design.

### EdU area proportion and EdU intensity analysis

Depending on the genetic background, a single confocal section or a substack of 3 sections was chosen within each disc, capturing the highest nuclear area of the disc proper. Intensity-based thresholding was used to generate a binary mask of nuclei (DAPI) and EdU areas (EdU). Masks were then expanded with the functions "fill holes" and "dilate". Pixel fluorescence intensities for all channels in these masks were obtained using the ‘Save XY Coordinates’ function, either on the whole tissue or in a ROI selected with the ‘Freehand Selection’ tool in Fiji. The percentage area of replicating DNA in the image was calculated as the ratio of the number of EdU-positive pixels in the DAPI mask over the number of pixels in the DAPI mask. This measure approximates the percentage of cells undergoing DNA replication, circumventing technical difficulties of segmenting individual nuclei in the tightly packed pseudostratified wing epithelium. The average intensity of EdU was calculated by averaging pixel intensities within the EdU mask only. If a stack of 3 section was used, only the EdU percentage was calculated.

### FUCCI-based cell cycle analysis

The proportion of cell-cycle phases (G1, S, LateS, G2) was calculated in Fiji/ImageJ using FUCCI-expressing imaginal discs. A DAPI mask was generated to analyze nuclear levels of FUCCI.NLS markers, by following the same steps described above. After intensity-based thresholding based on subtracting disc-specific non-nuclear background, the fluorescence intensities for each nuclear pixel coordinate was exported using the ‘save XY coordinates’ function. The pixel population was then divided into 4 cell-cycle phases: Ubi-GFP.E2f.1-230 positive area (G1), Ubi-mRFP1.NLS.CycB.1-266/CyO positive area (Late S), Ubi-GFP.E2f.1-230 and Ubi-mRFP1.NLS.CycB.1-266/CyO double positive area (G2), Ubi-GFP.E2f.1-230 and Ubi-mRFP1.NLS.CycB.1-266/CyO double negative area (S).

### γH2Av intensity analysis

A single confocal section was chosen which maximally captured the nuclear DAPI area of the disc proper. For *hid-*expressing discs, the proliferative ROI was created by free-hand selection of the *rn-*GAL4 defined pouch area. For *egr-*expressing discs, the proliferative ROI was created by (1) free-hand selection of the JNK-signaling area in the TRE-RFP channel and then (2) subtracting it from a free-hand selection of the pouch area using characteristic folding patterns as guides. This approach created a ROI of the proliferative domain and excluded the JNK-signaling area with G2-arrested cells and inflammatory signaling. Average intensity was measured for the DAPI channel and the γH2A channel in the ROI. All γH2A intensities were normalized by the DAPI intensity.

## Supporting information

S1 Fig Compensatory proliferation is associated with short G1, G2 and S-phases and EdU incorporation is not sensitive to tissue architecture defects. **(A-D)** Control wing disc (A,C) and wing disc after 8 h of *hid-*expression in the pouch domain (D,E) were assessed for DNA replication activity by EdU incorporation. Discs were stained with DAPI to visualize nuclei and were assessed for DNA replication activity by EdU incorporation. Pyknotic nuclei in (C) confirm onset of *hid*-induced cell death with the wing disc pouch domain where *hid* is expressed under the control of *rn*-GAL4 (cyan dotted line). S-phase-specific incorporation of the nucleotide analogue EdU into replicating DNA is already elevated in the pouch after 8 h of *hid*-expression (D). **(E,F)** Nota of control wing disc and (E) wing disc after 18 h of *hid-*expression in the pouch domain and at 6 h into the recovery period (F). Discs express two ‘flip-out’ construct to generate labelled clones, either controlling expression of GFP (green) or of Lac-Z (red). As both constructs are induced independently, clones either express GFP (green), LacZ (red) or both (yellow). **(G)** Quantification of number of cells per clone in the notum domain, from control or *hid-*expressing discs. Mean and 95% confidence interval (CI) are shown. Welch’s test was performed to test for statistical significance. (WT, n = 42 clones and Hid, n = 52 clones, ns p = 0.0747). **(H)** Peripodium of wild type wing was stained with DAPI to visualize nuclei (H), expresses the FUCCI reporter system, *ubi*-GFP-E2f1^1-230^ (green in overlay) and *ubi*-mRFP-NLS-CycB^1-266^ (red in overlay) (H’,H”,H”“,H”“‘). Discs were assessed for DNA replication activity by EdU incorporation (H”‘,H”“‘). (H”“) Composite view of H,H’,H”. (H”“‘) Composite view of H’,H”,H”‘. Euchromatin correlates with lower DAPI staining and is replicated early (magenta arrow). Satellite repeats (heterochromatin) correlate with bright DAPI staining and replicate late (purple arrow). **(I)** Wild type wing disc expressing the FUCCI reporter system, *ubi*-GFP-E2f1^1-230^ (green in overlay I,I”) and *ubi*-mRFP-NLS-CycB^1-266^ (red in overlay I,I”) and stained with DAPI to visualize nuclei. An extremely apical section through the wing pouch visualizes mitotic cells. Mitosis occurs exclusively on the apical surface in imaginal discs. Magnified view in I’ and I” is indicated by blue frame in I. DAPI staining visualizes progression through M-phase: metaphase plates (white arrows) and two separate nuclei (blue arrows). **(J)** Schematic representation of cell cycle phase identification using the FUCCI reporters validated by EdU incorporation assays and mitotic markers. **(K)** Flow cytometry analysis of DNA content in undamaged control wing discs (grey) and in wing disc after 24 h of *hid-*expression (red). The pouch of the wing disc was labeled by *rn-GAL4*-driven expression of *UAS-GFP* and thus cells outside the *rn-GAL4* domain can be distinguished by the lack of GFP expression. GFP-negative events were plotted. The cell cycle of cells outside of the control and *hid-*expressing domains in the pouch is not different. **(L)** Flow cytometry analysis of DNA content in undamaged control wing discs (grey) and in wing disc after 24 h of *hid-*expression (green in I, red in I’), as shown in Fig 1O. The gating of Hoechst-channel (DNA) was opened (if compared to Fig 1O) to also visualize flow cytometry events with higher Hoechst intensity. No difference in the proportion of these higher intensity events between control and *hid-*expressing discs can be detected. This suggests that *hid-*expressing discs do not experience a specific increase in events that may represent endoreplicating nuclei. **(M-Q)** Control wing disc (M,P) and wing disc after 24 h of ectopic MMP1 (N), MMP2 (M) and *cora*-RNAi (Q) expression in the pouch domain. Discs were stained with DAPI to visualize nuclei and were assessed for DNA replication activity by BrDU incorporation or EdU incorporation. Cora-RNAi expressing discs were stained for Cora to assess knock-down efficiency. Maximum projection of the apical domain is shown (P’,Q’). Maximum projections of multiple confocal sections are shown in (B,D); Single confocal sections are shown in (A,C,E,F,H,I). Maximum projection of the apical domain is shown (P’,Q’). Scale bars: 50 μm.
(PDF)

S2 Fig EdU incorporation is not sensitive to tissue architecture defects. **(A)** Wing disc after 24 h of *egr-*expression (E) in the pouch domain. Discs also express the JNK-reporter TRE-RFP (A‘, red in A”“). The disc was stained with DAPI to visualize nuclei (A, red in A”“‘), for the mitotic marker phospho-His3 to visualize M-phase cells (A”‘, green) and was assessed for DNA replication activity by EdU incorporation (grey). Compare number of phospho-His3 positive events to the number of EdU labelled nuclei to estimate relatively low frequency of M-phase cells in discs. **(B-E)** Nota of control wing disc (B,D) and and wing disc after 24 h of *hid-*expression (C) and after 24 h of *egr-*expression (E) in the pouch domain. Discs were stained with DAPI to visualize nuclei and were assessed for DNA replication activity by EdU incorporation (B’-E’). Cyan star in (E) marks small domain of frequent transdetermination as described in M. I. Worley, L. A. Alexander and I. K. Hariharan, CtBP impedes JNK- and Upd/STAT-driven cell fate misspecifications in regenerating Drosophila imaginal discs, Elife 2018 Vol. 7. Cells in this patch undergo compensatory-like proliferation as part of the transdetermination program and therefore incorporate more EdU. Scale bars: 50 μm.
(PDF)

S3 Fig Compensatory proliferation does not revert to a developmentally younger cell cycle and JNK signaling cannot cell-autonomously promote cell cycle acceleration. **(A-E)** Wing discs at different developmental stages (day 5, 6, 7 and 8 after egg lay)(A-D), and a wing disc after 24 h of *hid-*expression (E). Discs were stained with DAPI to visualize nuclei and were assessed for DNA replication activity to visualize S-phase cells by EdU incorporation. Please compare (A-D) to (E). **(F-I)** Wing disc expressing the FUCCI reporter system, *ubi*-GFP-E2f1^1-230^ (green in overlay) and *ubi*-mRFP-NLS-CycB^1-266^ (red in overlay) at different developmental stages (day 5, 6, 7 and 8 after egg lay). Discs were stained with DAPI to visualize nuclei. **(J)** Quantification of cell cycle phase distribution using the FUCCI profile at day 5 and day 8 after egg lay. Phases were defined as described in experimental procedures. A Welch’s test was performed to test for statistical significance between day 5 and day 8 wing discs: G1 (p = 0.0269 *), S (p = 0.3828 ns), Late S (p = 0.9363 ns), G2 (p = 0.0092 **). n = 5 disc for each day. **(K-M)** Control wing disc (K) and wing disc after 24 h of *hid-*expression in the pouch domain (L, M). Discs were stained with DAPI to visualize nuclei. Discs were assessed for JNK activity by TRE-RFP reporter activity (K,L). Discs were assessed for DNA replication activity by EdU incorporation (M). **(N,O)** Control wing disc (N) and wing disc after 12 h of *puc*-RNAi*-*expression in the pouch domain (O). Discs were stained with DAPI to visualize nuclei. Basal section of the disc from Fig 3D–3E are shown. Pyknotic nuclei visualize cell death patterns and indicate that, as expected, JNK-activity is elevated upon knock-down of *puc*. **(P)** Wing disc after 24 h of *bsk*^*DN*^*-*expression in the *engrailed* domain using *en*-GAL4 (P’, red in P”‘). Discs were stained with DAPI to visualize nuclei (P). Discs were assessed for DNA replication activity by EdU incorporation (P”, cyan in P”‘). **(Q, R)** Control wing disc (Q) and wing disc after 24 h of co-expressing *hid* and *bskDN* in the pouch domain (R). Discs were stained with DAPI to visualize nuclei (Q,R). Discs were assessed for DNA replication activity by EdU incorporation (Q’,R’). Graphs display mean and 95% confidence interval (CI). Single confocal sections are shown. Scale bars: 50 μm. Dotted lines (red) outline stereotypic folds in the wing discs.
(PDF)

S4 Fig Yorkie activity and ERK signaling are elevated in proliferating cells of *hid-*expressing discs. **(A-D)** Control wing disc (A,C), wing disc after 24 h of *hid-*expression in the pouch domain (B,D). Discs either express Yorkie-GFP (A,B) (green) or the ERK reporter miniCic-mCherry (C,D) (green). Discs were stained with DAPI to visualize nuclei and were assessed for DNA replication activity by EdU incorporation (A’-D’, or magenta). Magnified view of the pouch domain shown. **(E, F)** Control wing disc (E) and wing disc after 24 h of *hid-*expression in the pouch domain (F). Discs express the JAK/STAT reporter *10xStat92E>dGFP* (green) and DNA replication activity was assessed by EdU incorporation (magenta). Magnified view of the pouch domain shown (E”,F”). Same disc as in Fig 4G and 4H are shown. Maximum projections of multiple confocal sections are shown in (E,F); single sections are shown in (A,B,C,D). Scale bars: 50 μm.
(PDF)

S5 Fig JAK/STAT signaling and Myc-expression are elevated in proliferating cells of *egr-*expressing discs and signaling signature is still the same 24 h into the recovery period. **(A-D)** Control wing disc (A,C), wing disc after 24 h of *egr-*expression in the pouch domain (B,D). Discs either express Yorkie-GFP (A,B) (green) or the ERK reporter miniCic-mCherry (C,D) (green). Discs were stained with DAPI to visualize nuclei (A”‘-D”‘) and were assessed for DNA replication activity by EdU incorporation (A’-D’ or magenta). Magnified view of the pouch domain shown. **(E,F)** Control wing disc (E) and wing disc after 24 h of *egr-*expression in the pouch domain (F). Discs express the JAK/STAT reporter *10xStat92E>dGFP* (green) and DNA replication activity was assessed by EdU incorporation (magenta). Magnified view of the pouch domain (E”,F”). Please note that these discs are the same as shown in Figs 5E and 4G. **(G-P)** Control wing disc (G,I,K,M,O), or wing disc after 24 h of expression in the pouch domain and then analyzed 24 h into the recovery period, after *egr*-expression (H,J,L,N) or hid-expression (P) was stopped. Discs either express Yorkie-GFP (G,H), the ERK reporter miniCic-mCherry (I,J), the JAK/STAT reporter *10xStat92E>dGFP* (K,L) or an endogenously tagged Myc-GFP construct (M-P). Images with increased brightness show the presence of Myc-GFP in the regenerative domain (M”,N”). We suggest that the Myc-expressing cells in the anterior pouch domain of control disc are killed by *egr*-expression and a new expression pattern of Myc is set up *de novo* by tissue damage signals, which is maintained throughout the regenerative period. The interspersed apoptosis and the lack of a JNK-driven wound response program in hid-expressing disc maintains the original myc-expression pattern in the anterior pouch. Discs were stained with DAPI to visualize nuclei. Maximum projections of multiple confocal sections are shown in (E,F); single sections are shown in (A-D). Scale bars: 50 μm. Scale bars: 50 μm.
(PDF)

S6 Fig Yki and ERK cooperate to drive compensatory proliferation in response to non-inflammatory damage. **(A,B)** A wing disc expressing the act-GAL4 ‘flip-out’ system controlling the mosaic expression of GFP (A’,B’, or green) and UAS-Warts-RNAi (A), or UAS-Hippo-RNAi (B). Discs were stained with DAPI to visualize nuclei (A,B) and were assessed for DNA replication activity by EdU incorporation (A”,B”, or magenta). **(C,D)** Control wing disc (C), and a wing disc heterozygous for *yki*^*B5*^ (D). Discs were stained with DAPI to visualize nuclei (C,D) and were assessed for DNA replication activity by EdU incorporation (C’,D’). **(E-H)** Control wing disc (E), and a control wing disc after 24 h of *Egfr*.*DN*-expression in the pouch domain (F). A control wing disc after 24 h of *hid-*expression (G) and a wing disc after 24 h of *hid-* and *Egfr*.*DN*-co-expression in the pouch domain (H). Discs were stained with DAPI to visualize nuclei and were assessed for DNA replication activity by EdU incorporation. Egfr.DN does not change EdU incorporation dynamics in wild type and *hid*-expressing discs. **(I-L)** Control wing disc (I) and a control wing disc heterozygous for *Ras*^*1*^ (J). A control wing disc after 24 h of *hid-*expression (K) and a wing disc after 24 h of *hid-*expression and heterozygous for *Ras*^*1*^ (L). Discs were stained with DAPI to visualize nuclei and were assessed for DNA replication activity by EdU incorporation. Heterozygosity for *Ras*^*1*^ does not change EdU incorporation dynamics in wild type and *hid*-expressing discs. **(M)** Quantification of the percentage of DAPI areas that were positive for incorporated EdU in *hid-*expressing discs and *hid-*expressing discs heterozygous for *Ras*^*1*^. This serves as a proxy for the number of nuclei undergoing DNA replication. Mean and 95% CI are shown. Welch’s test was performed to test for statistical significance. (Hid, n = 8 discs; Hid, Ras^1/+^, n = 7 discs, ns, p = 0.266). **(N)** Quantification of incorporated EdU, measured as the mean EdU intensity in the EdU area within the pouch of *hid-*expressing discs and *hid-*expressing discs heterozygous for *Ras*^*1*^. This serves as a proxy for the dynamics of nucleotide incorporation. A Welch’s test was performed to test for statistical significance. (Hid, n = 8 discs, Hid, Ras^1/+^, n = 7 discs, ns, p = 0.255). Single sections are shown in (A-F,I-L). Maximum projections of multiple confocal sections are shown in (G,H). Scale bars: 50 μm.
(PDF)

S7 Fig Analysis of necessity of Stat92E for accelerated nucleotide incorporation. **(A,B)** Control wing disc after 24 h of *egr-*expression (A) and a wing disc heterozygous for the *Stat92E*^*85C3*^ null allele after 24 h of *egr-*expression in the pouch domain (B). Discs were stained with DAPI to visualize nuclei and were assessed for DNA replication activity by EdU incorporation.
(PDF)

S8 Fig Insulin signaling is sufficient to drive accelerated DNA replication. **(A,B)** Control wing disc (A), wing disc after 24 h of UAS-InR-DA expression in the pouch domain (B). Discs were stained with DAPI to visualize nuclei and were assessed for DNA replication activity by EdU incorporation. **(C)** Quantification of the percentage of DAPI areas that were positive for incorporated EdU in control wing discs or UAS-InR-DA expressing wing discs. This serves as a proxy for the number of nuclei undergoing DNA replication (WT, n = 9 discs, UAS-InR-DA, n = 9 discs, p = 0.1711). **(C’)** Quantification of incorporated EdU, measured as mean EdU intensity in the EdU area within the pouch. A Welch’s test was performed to test for statistical significance. (WT, n = 9 discs, UAS-InR-DA, n = 9 discs, *p = 0.0187). Single sections are shown in (A,B). Scale bars: 50 μm.
(PDF)

S1 Table Fly strains used in this study. (DOCX)
