# Supplementary figures and images for: Distinct signaling signatures drive compensatory proliferation via S-phase acceleration
Source: PLoS Genet. 2022 Dec 15;18(12):e1010516. doi: 10.1371/journal.pgen.1010516 (PMC9799308; doi:10.1371/journal.pgen.1010516)

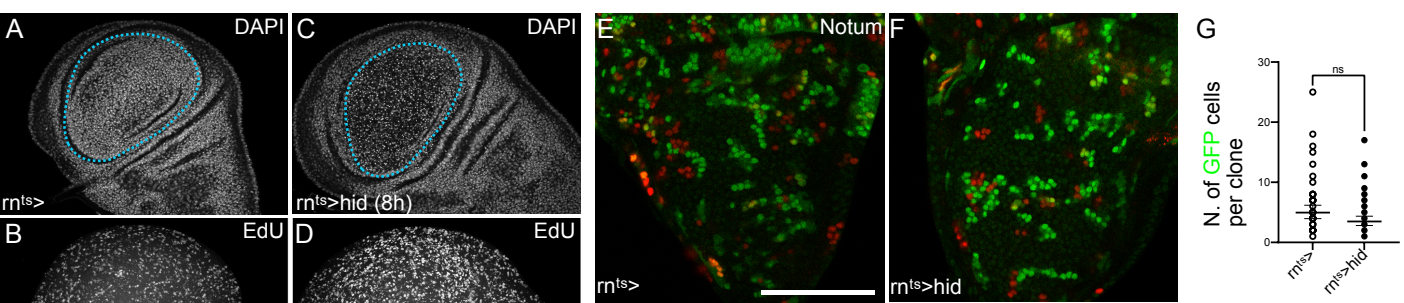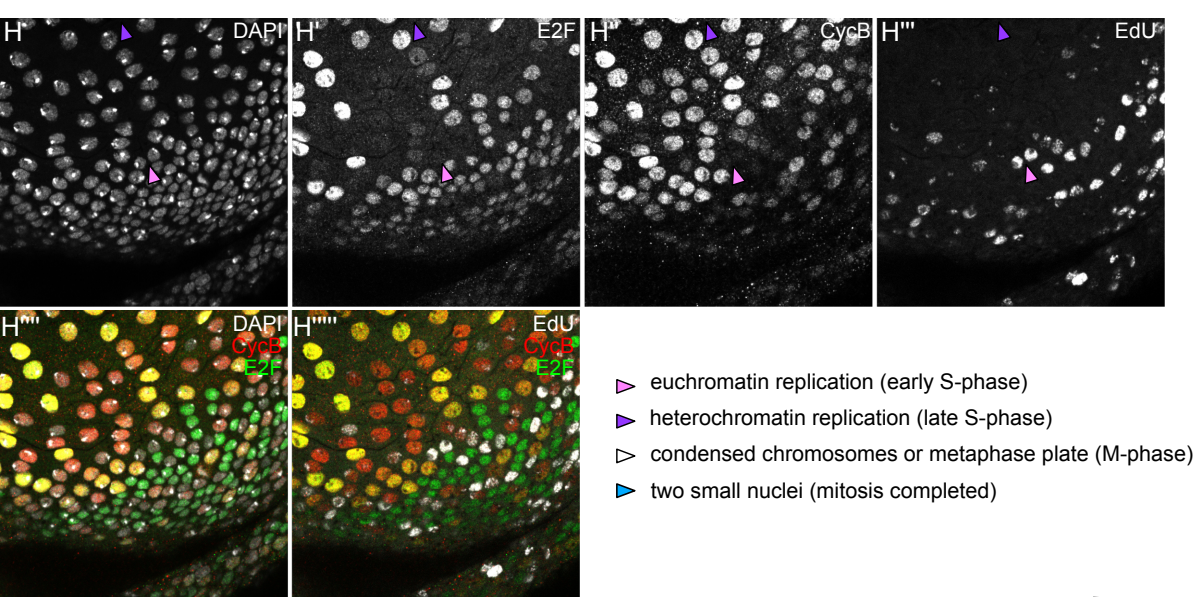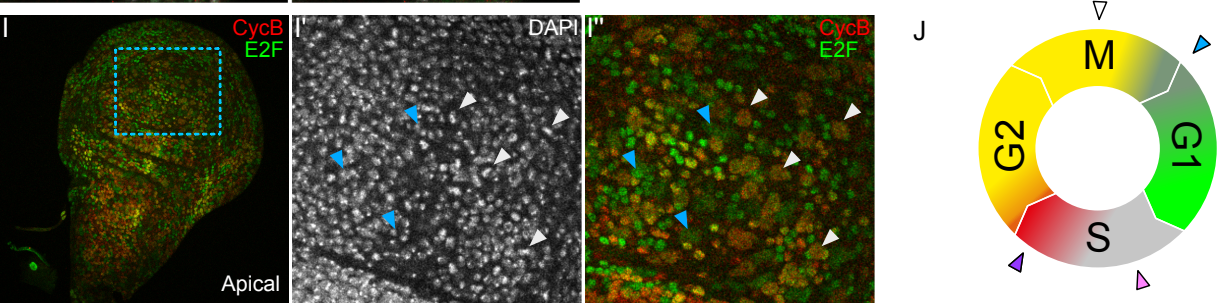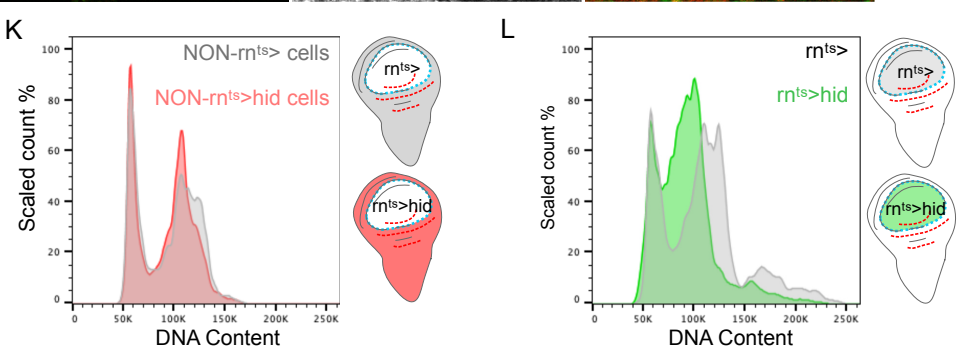

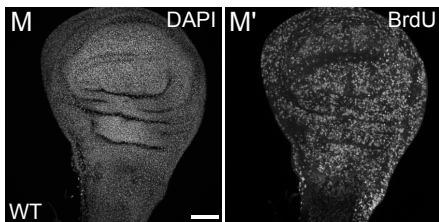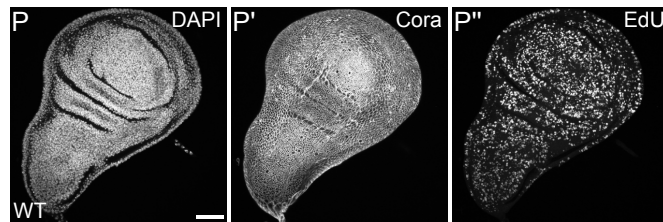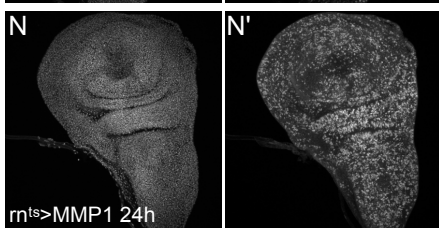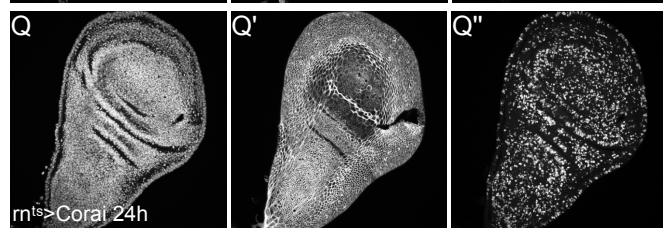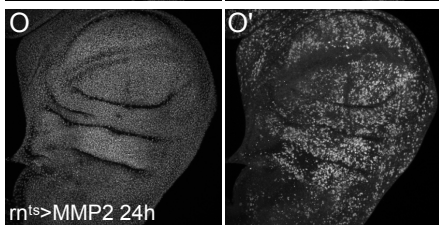

Supplement: S1 Fig — (A-D) Control wing disc (A,C) and wing disc after 8 h of hid-expression in the pouch domain (D,E) were assessed for DNA replication activity by EdU incorporation. Discs were stained with DAPI to visualize nuclei and were assessed for DNA replication activity by EdU incorporation. Pyknotic nuclei in (C) confirm onset of hid-induced cell death with the wing disc pouch domain where hid is expressed under the control of rn-GAL4 (cyan dotted line). S-phase-specific incorporation of the nucleotide analogue EdU into replicating DNA is already elevated in the pouch after 8 h of hid-expression (D). (E,F) Nota of control wing disc and (E) wing disc after 18 h of hid-expression in the pouch domain and at 6 h into the recovery period (F). Discs express two ‘flip-out’ construct to generate labelled clones, either controlling expression of GFP (green) or of Lac-Z (red). As both constructs are induced independently, clones either express GFP (green), LacZ (red) or both (yellow). (G) Quantification of number of cells per clone in the notum domain, from control or hid-expressing discs. Mean and 95% confidence interval (CI) are shown. Welch’s test was performed to test for statistical significance. (WT, n = 42 clones and Hid, n = 52 clones, ns p = 0.0747). (H) Peripodium of wild type wing was stained with DAPI to visualize nuclei (H), expresses the FUCCI reporter system, ubi-GFP-E2f11-230 (green in overlay) and ubi-mRFP-NLS-CycB1-266 (red in overlay) (H’,H”,H”“,H”“‘). Discs were assessed for DNA replication activity by EdU incorporation (H”‘,H”“‘). (H”“) Composite view of H,H’,H”. (H”“‘) Composite view of H’,H”,H”‘. Euchromatin correlates with lower DAPI staining and is replicated early (magenta arrow). Satellite repeats (heterochromatin) correlate with bright DAPI staining and replicate late (purple arrow). (I) Wild type wing disc expressing the FUCCI reporter system, ubi-GFP-E2f11-230 (green in overlay I,I”) and ubi-mRFP-NLS-CycB1-266 (red in overlay I,I”) and stained with DAPI to [file pgen.1010516.s001.pdf]

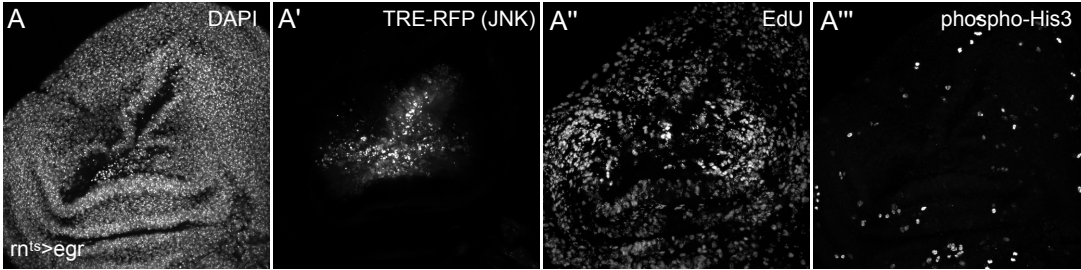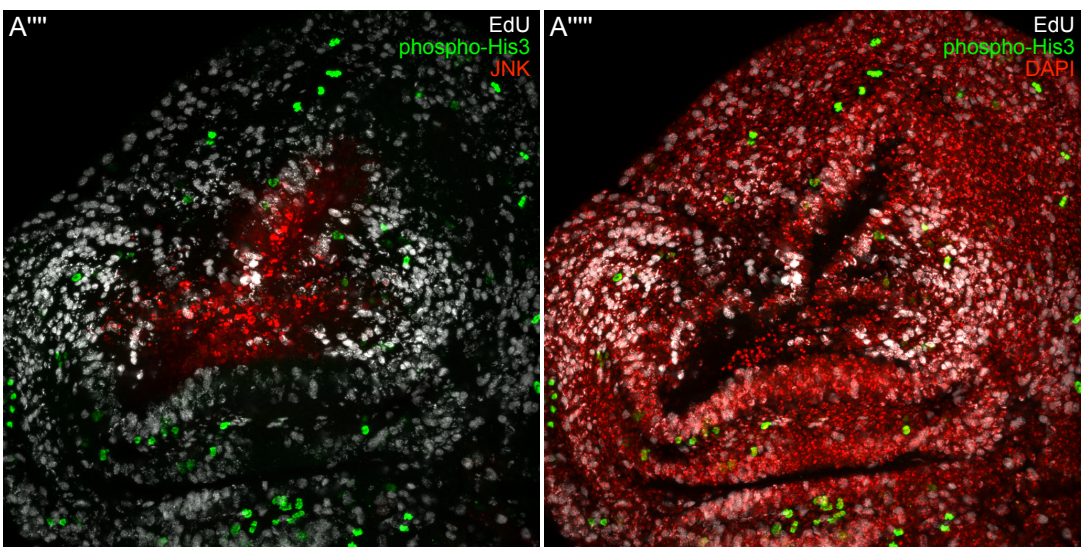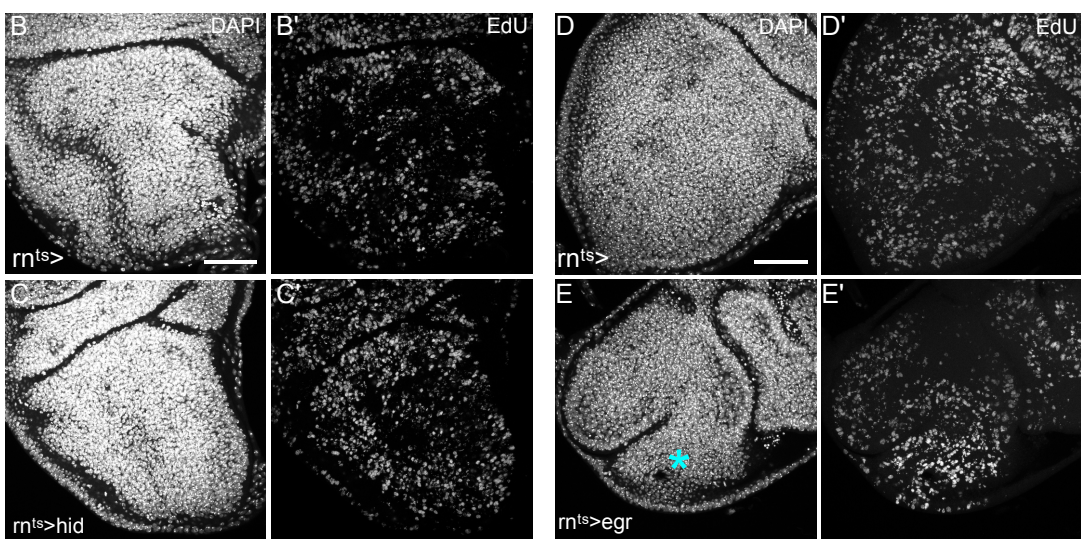

Supplement: S2 Fig — (A) Wing disc after 24 h of egr-expression (E) in the pouch domain. Discs also express the JNK-reporter TRE-RFP (A‘, red in A”“). The disc was stained with DAPI to visualize nuclei (A, red in A”“‘), for the mitotic marker phospho-His3 to visualize M-phase cells (A”‘, green) and was assessed for DNA replication activity by EdU incorporation (grey). Compare number of phospho-His3 positive events to the number of EdU labelled nuclei to estimate relatively low frequency of M-phase cells in discs. (B-E) Nota of control wing disc (B,D) and and wing disc after 24 h of hid-expression (C) and after 24 h of egr-expression (E) in the pouch domain. Discs were stained with DAPI to visualize nuclei and were assessed for DNA replication activity by EdU incorporation (B’-E’). Cyan star in (E) marks small domain of frequent transdetermination as described in M. I. Worley, L. A. Alexander and I. K. Hariharan, CtBP impedes JNK- and Upd/STAT-driven cell fate misspecifications in regenerating Drosophila imaginal discs, Elife 2018 Vol. 7. Cells in this patch undergo compensatory-like proliferation as part of the transdetermination program and therefore incorporate more EdU. Scale bars: 50 μm. (PDF) [file pgen.1010516.s002.pdf]

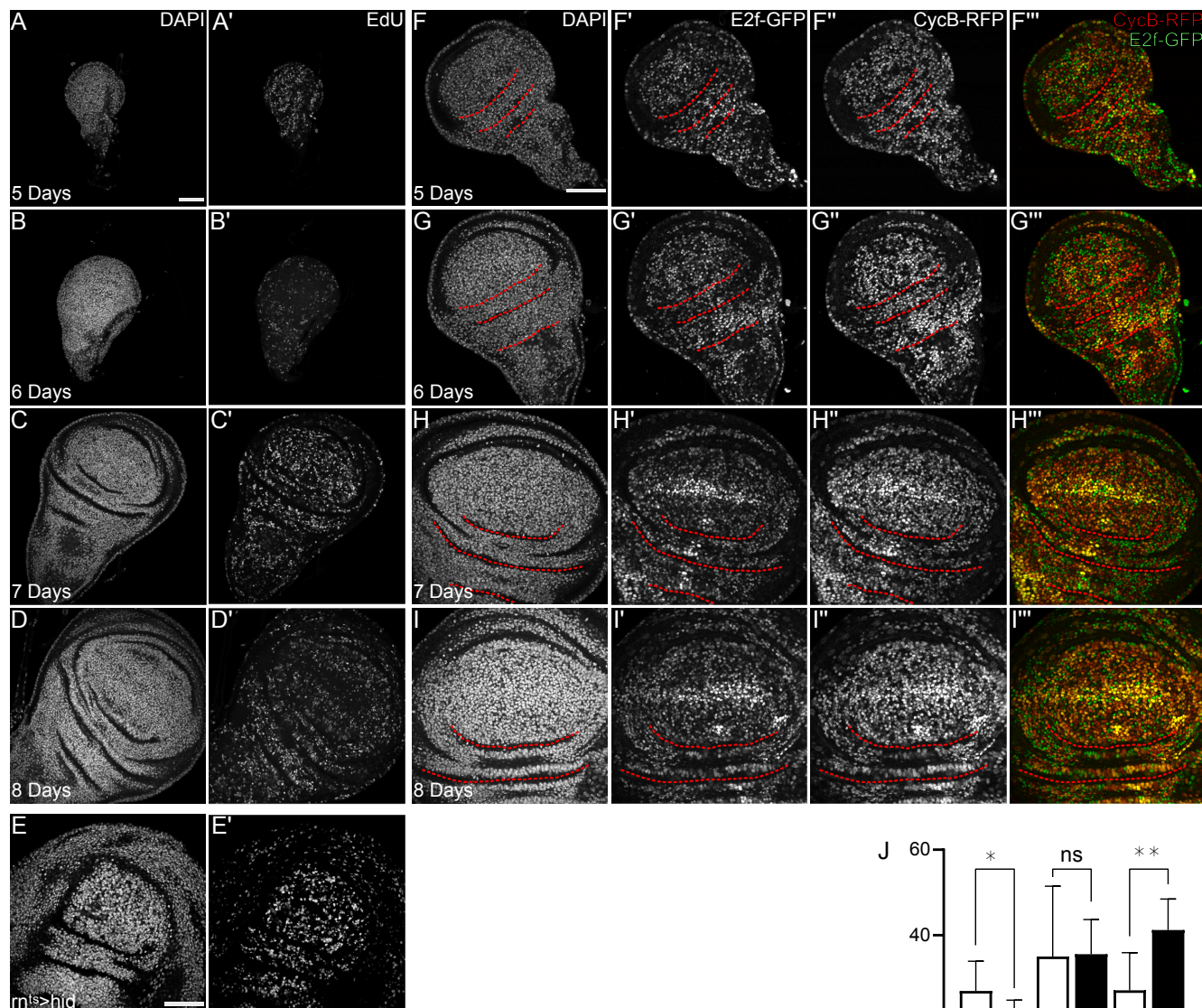

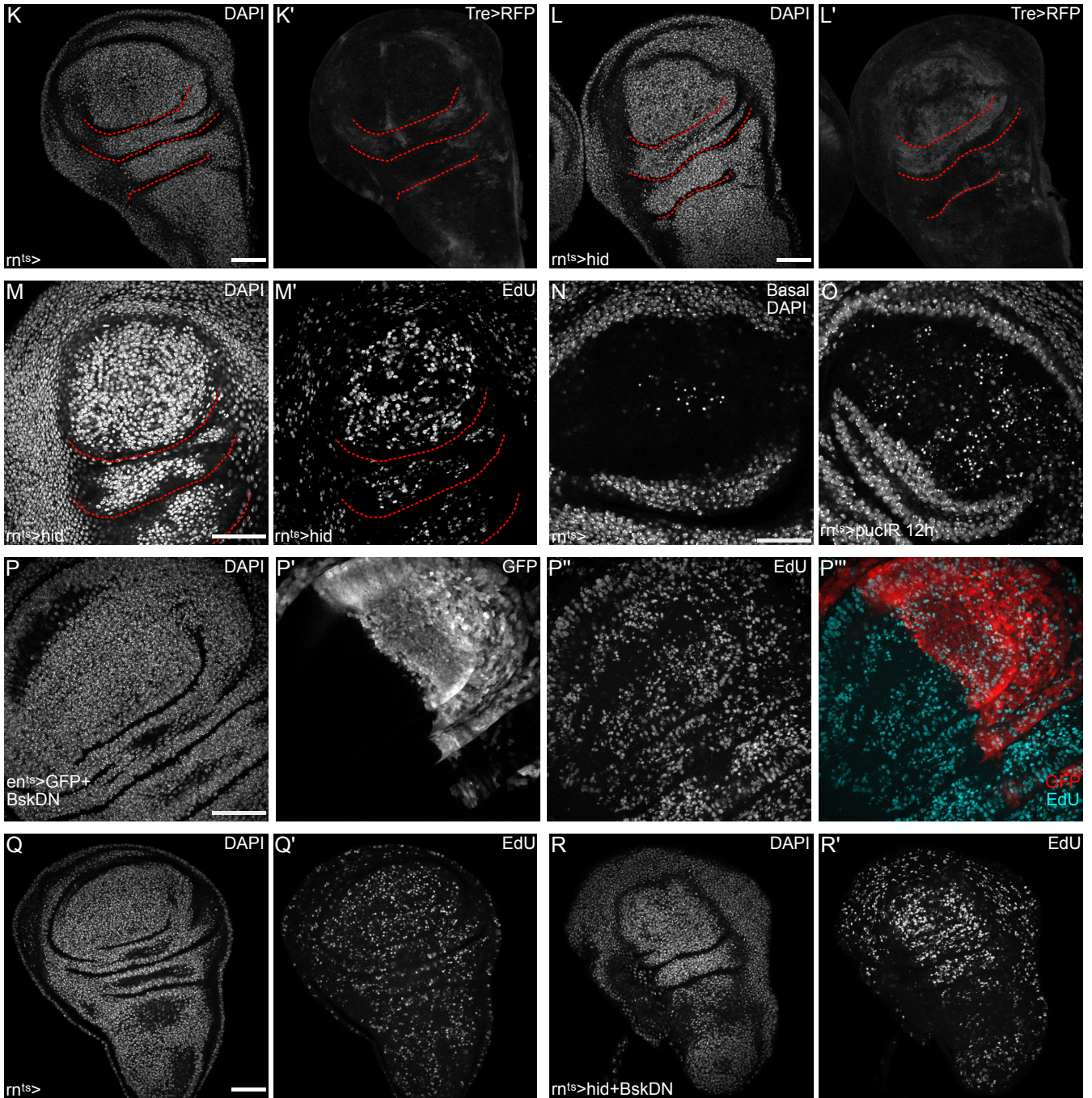

Supplement: S3 Fig — (A-E) Wing discs at different developmental stages (day 5, 6, 7 and 8 after egg lay)(A-D), and a wing disc after 24 h of hid-expression (E). Discs were stained with DAPI to visualize nuclei and were assessed for DNA replication activity to visualize S-phase cells by EdU incorporation. Please compare (A-D) to (E). (F-I) Wing disc expressing the FUCCI reporter system, ubi-GFP-E2f11-230 (green in overlay) and ubi-mRFP-NLS-CycB1-266 (red in overlay) at different developmental stages (day 5, 6, 7 and 8 after egg lay). Discs were stained with DAPI to visualize nuclei. (J) Quantification of cell cycle phase distribution using the FUCCI profile at day 5 and day 8 after egg lay. Phases were defined as described in experimental procedures. A Welch’s test was performed to test for statistical significance between day 5 and day 8 wing discs: G1 (p = 0.0269 *), S (p = 0.3828 ns), Late S (p = 0.9363 ns), G2 (p = 0.0092 **). n = 5 disc for each day. (K-M) Control wing disc (K) and wing disc after 24 h of hid-expression in the pouch domain (L, M). Discs were stained with DAPI to visualize nuclei. Discs were assessed for JNK activity by TRE-RFP reporter activity (K,L). Discs were assessed for DNA replication activity by EdU incorporation (M). (N,O) Control wing disc (N) and wing disc after 12 h of puc-RNAi-expression in the pouch domain (O). Discs were stained with DAPI to visualize nuclei. Basal section of the disc from Fig 3D–3E are shown. Pyknotic nuclei visualize cell death patterns and indicate that, as expected, JNK-activity is elevated upon knock-down of puc. (P) Wing disc after 24 h of bskDN-expression in the engrailed domain using en-GAL4 (P’, red in P”‘). Discs were stained with DAPI to visualize nuclei (P). Discs were assessed for DNA replication activity by EdU incorporation (P”, cyan in P”‘). (Q, R) Control wing disc (Q) and wing disc after 24 h of co-expressing hid and bskDN in the pouch domain (R). Discs were stained with DAPI to visualize nuclei (Q,R). Discs were ass [file pgen.1010516.s003.pdf]

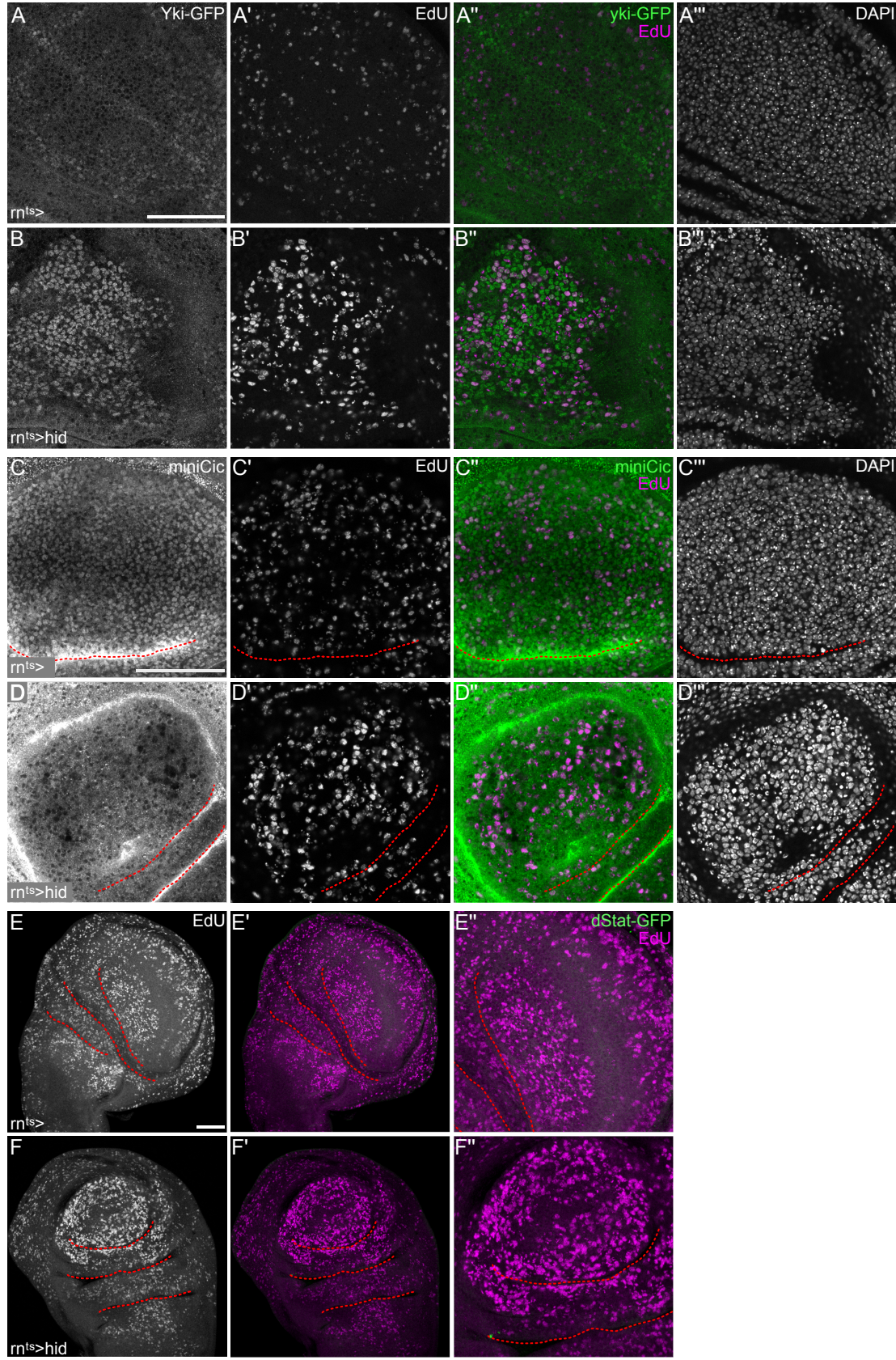

Supplement: S4 Fig — (A-D) Control wing disc (A,C), wing disc after 24 h of hid-expression in the pouch domain (B,D). Discs either express Yorkie-GFP (A,B) (green) or the ERK reporter miniCic-mCherry (C,D) (green). Discs were stained with DAPI to visualize nuclei and were assessed for DNA replication activity by EdU incorporation (A’-D’, or magenta). Magnified view of the pouch domain shown. (E, F) Control wing disc (E) and wing disc after 24 h of hid-expression in the pouch domain (F). Discs express the JAK/STAT reporter 10xStat92E>dGFP (green) and DNA replication activity was assessed by EdU incorporation (magenta). Magnified view of the pouch domain shown (E”,F”). Same disc as in Fig 4G and 4H are shown. Maximum projections of multiple confocal sections are shown in (E,F); single sections are shown in (A,B,C,D). Scale bars: 50 μm. (PDF) [file pgen.1010516.s004.pdf]

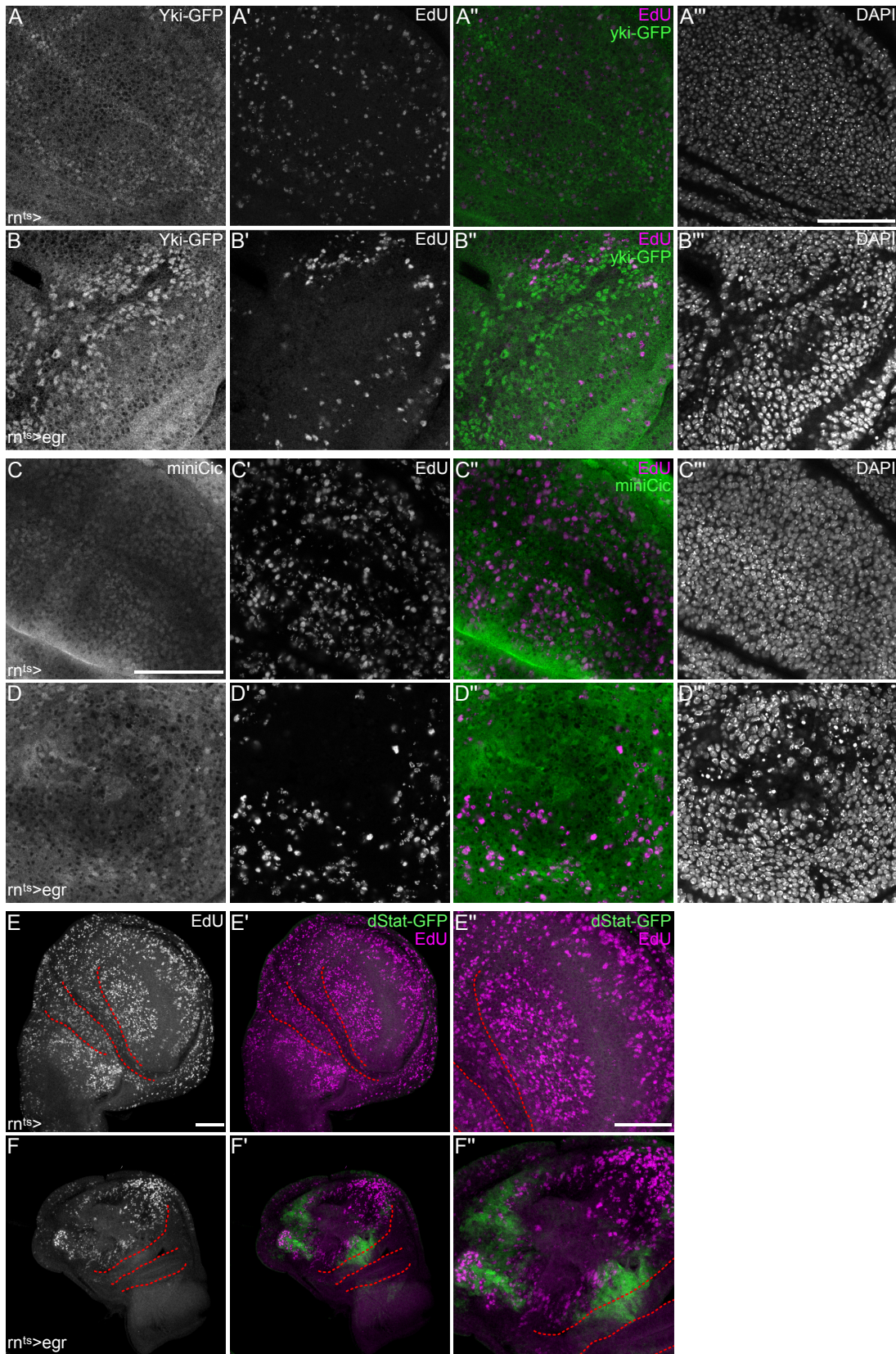

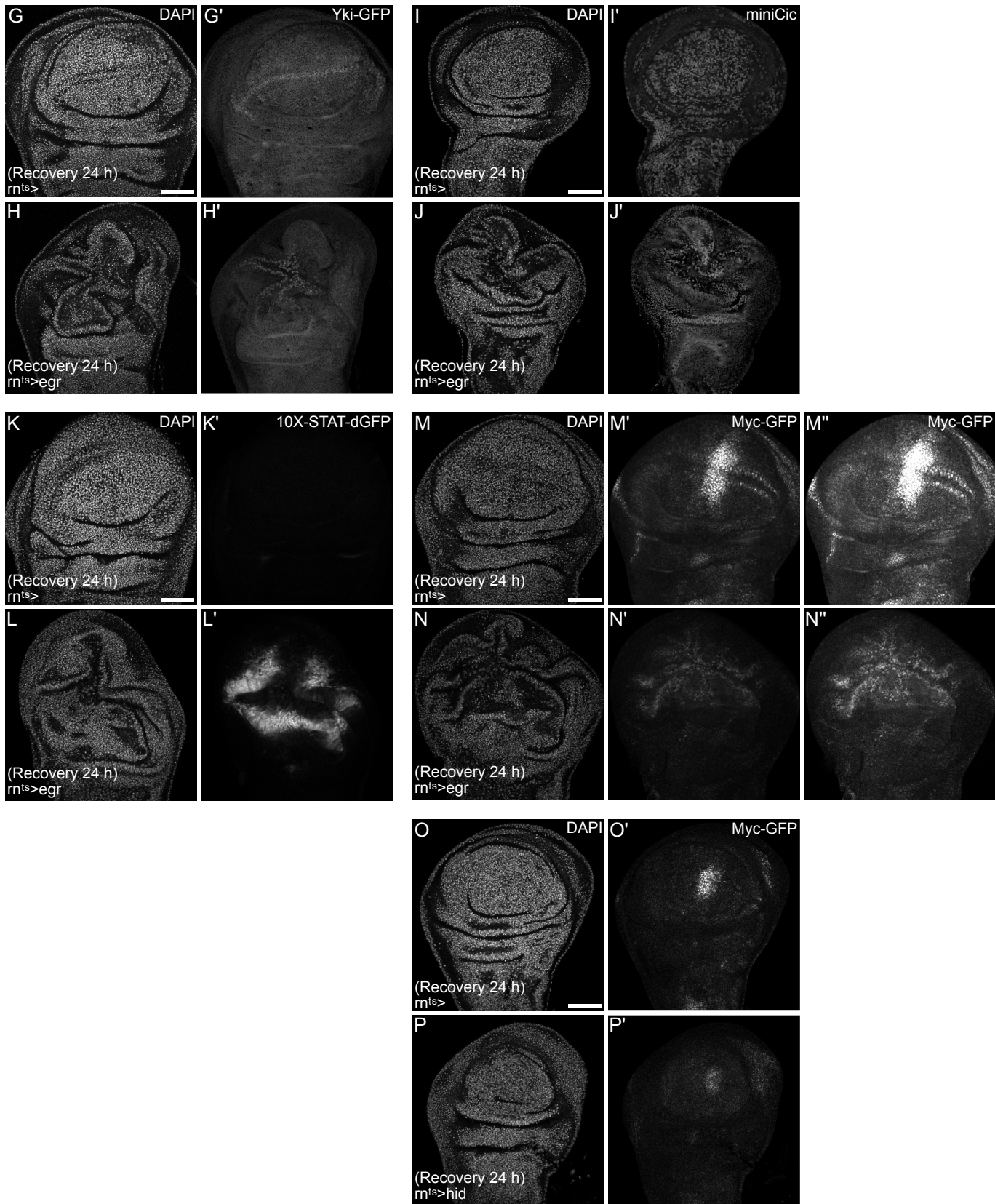

Supplement: S5 Fig — (A-D) Control wing disc (A,C), wing disc after 24 h of egr-expression in the pouch domain (B,D). Discs either express Yorkie-GFP (A,B) (green) or the ERK reporter miniCic-mCherry (C,D) (green). Discs were stained with DAPI to visualize nuclei (A”‘-D”‘) and were assessed for DNA replication activity by EdU incorporation (A’-D’ or magenta). Magnified view of the pouch domain shown. (E,F) Control wing disc (E) and wing disc after 24 h of egr-expression in the pouch domain (F). Discs express the JAK/STAT reporter 10xStat92E>dGFP (green) and DNA replication activity was assessed by EdU incorporation (magenta). Magnified view of the pouch domain (E”,F”). Please note that these discs are the same as shown in Figs 5E and 4G. (G-P) Control wing disc (G,I,K,M,O), or wing disc after 24 h of expression in the pouch domain and then analyzed 24 h into the recovery period, after egr-expression (H,J,L,N) or hid-expression (P) was stopped. Discs either express Yorkie-GFP (G,H), the ERK reporter miniCic-mCherry (I,J), the JAK/STAT reporter 10xStat92E>dGFP (K,L) or an endogenously tagged Myc-GFP construct (M-P). Images with increased brightness show the presence of Myc-GFP in the regenerative domain (M”,N”). We suggest that the Myc-expressing cells in the anterior pouch domain of control disc are killed by egr-expression and a new expression pattern of Myc is set up de novo by tissue damage signals, which is maintained throughout the regenerative period. The interspersed apoptosis and the lack of a JNK-driven wound response program in hid-expressing disc maintains the original myc-expression pattern in the anterior pouch. Discs were stained with DAPI to visualize nuclei. Maximum projections of multiple confocal sections are shown in (E,F); single sections are shown in (A-D). Scale bars: 50 μm. Scale bars: 50 μm. (PDF) [file pgen.1010516.s005.pdf]

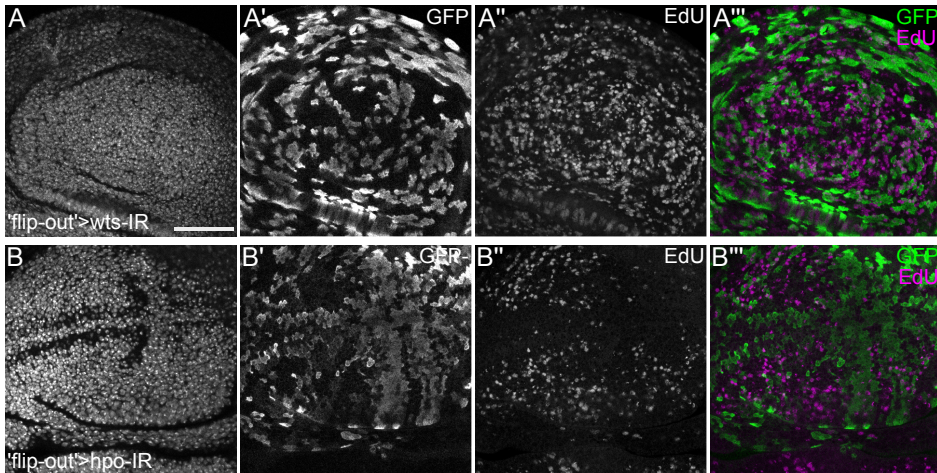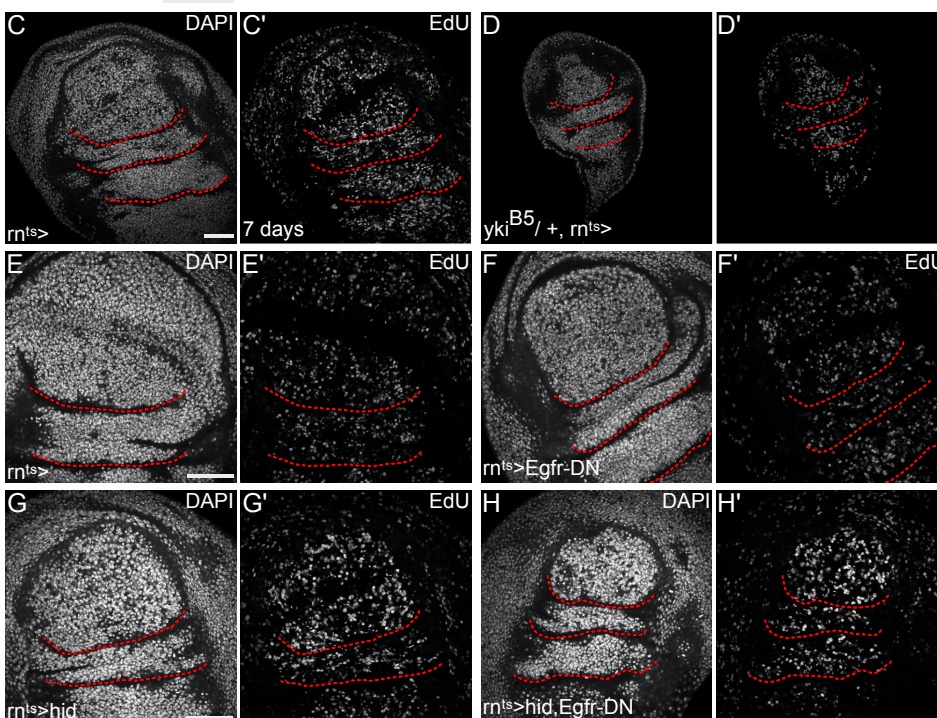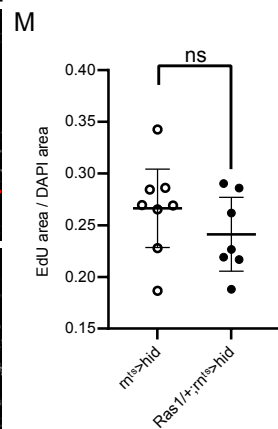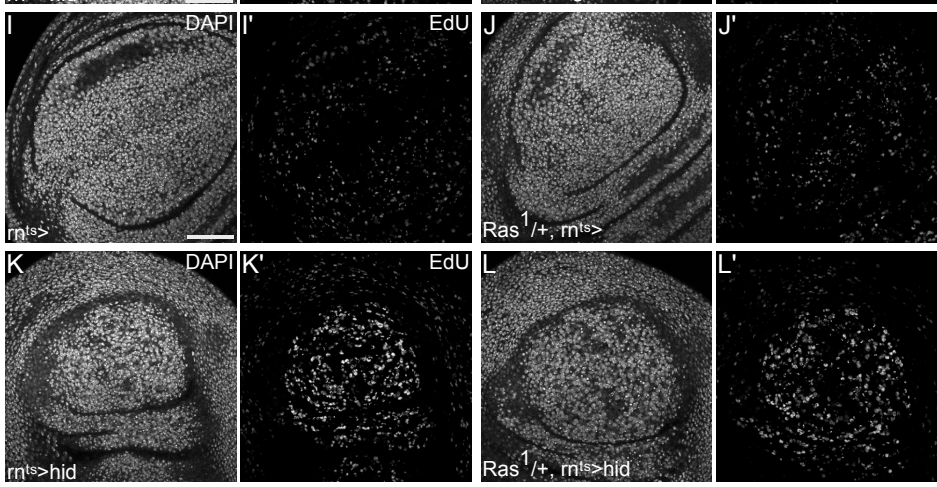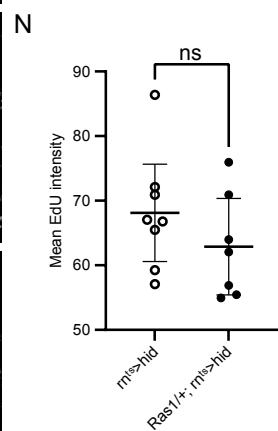

Supplement: S6 Fig — (A,B) A wing disc expressing the act-GAL4 ‘flip-out’ system controlling the mosaic expression of GFP (A’,B’, or green) and UAS-Warts-RNAi (A), or UAS-Hippo-RNAi (B). Discs were stained with DAPI to visualize nuclei (A,B) and were assessed for DNA replication activity by EdU incorporation (A”,B”, or magenta). (C,D) Control wing disc (C), and a wing disc heterozygous for ykiB5 (D). Discs were stained with DAPI to visualize nuclei (C,D) and were assessed for DNA replication activity by EdU incorporation (C’,D’). (E-H) Control wing disc (E), and a control wing disc after 24 h of Egfr.DN-expression in the pouch domain (F). A control wing disc after 24 h of hid-expression (G) and a wing disc after 24 h of hid- and Egfr.DN-co-expression in the pouch domain (H). Discs were stained with DAPI to visualize nuclei and were assessed for DNA replication activity by EdU incorporation. Egfr.DN does not change EdU incorporation dynamics in wild type and hid-expressing discs. (I-L) Control wing disc (I) and a control wing disc heterozygous for Ras1 (J). A control wing disc after 24 h of hid-expression (K) and a wing disc after 24 h of hid-expression and heterozygous for Ras1 (L). Discs were stained with DAPI to visualize nuclei and were assessed for DNA replication activity by EdU incorporation. Heterozygosity for Ras1 does not change EdU incorporation dynamics in wild type and hid-expressing discs. (M) Quantification of the percentage of DAPI areas that were positive for incorporated EdU in hid-expressing discs and hid-expressing discs heterozygous for Ras1. This serves as a proxy for the number of nuclei undergoing DNA replication. Mean and 95% CI are shown. Welch’s test was performed to test for statistical significance. (Hid, n = 8 discs; Hid, Ras1/+, n = 7 discs, ns, p = 0.266). (N) Quantification of incorporated EdU, measured as the mean EdU intensity in the EdU area within the pouch of hid-expressing discs and hid-expressing discs heterozygous for Ras1. This serves as a proxy [file pgen.1010516.s006.pdf]

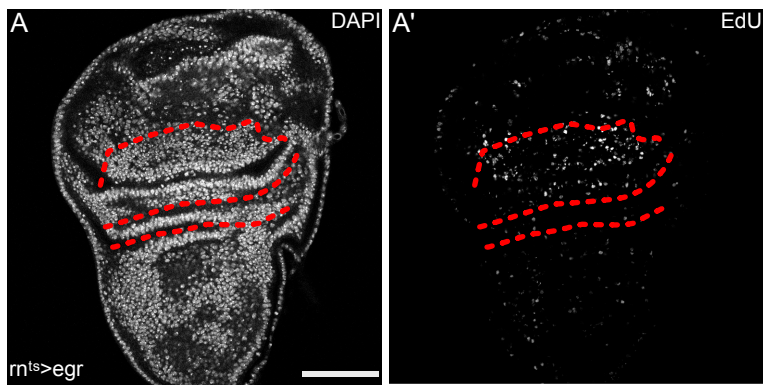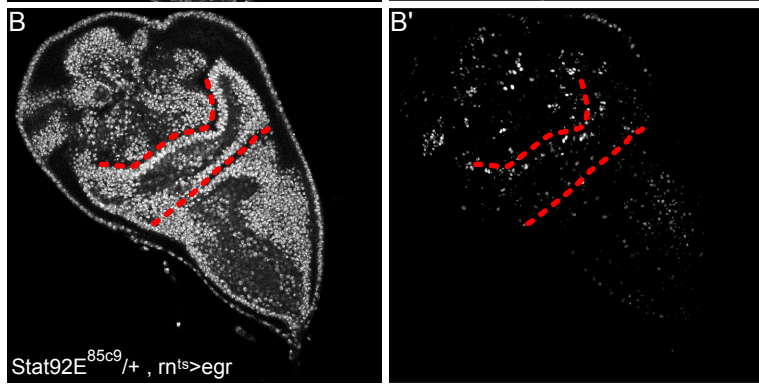

Supplement: S7 Fig — (A,B) Control wing disc after 24 h of egr-expression (A) and a wing disc heterozygous for the Stat92E85C3 null allele after 24 h of egr-expression in the pouch domain (B). Discs were stained with DAPI to visualize nuclei and were assessed for DNA replication activity by EdU incorporation. (PDF) [file pgen.1010516.s007.pdf]

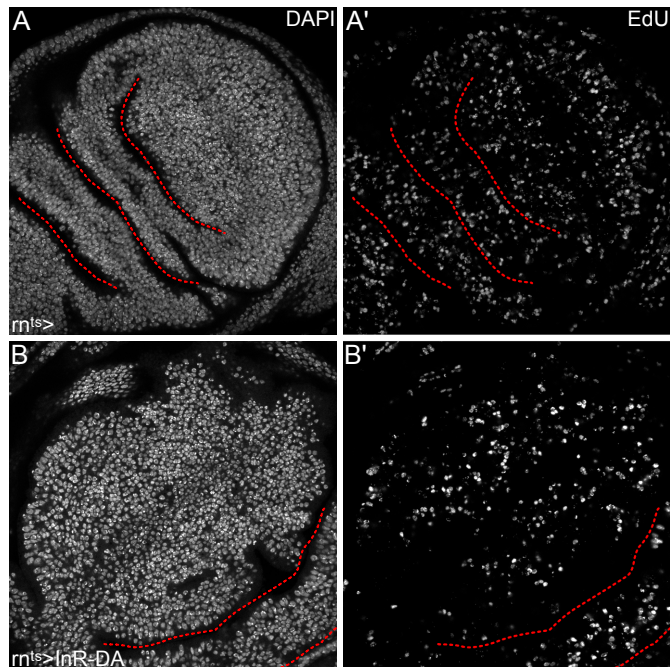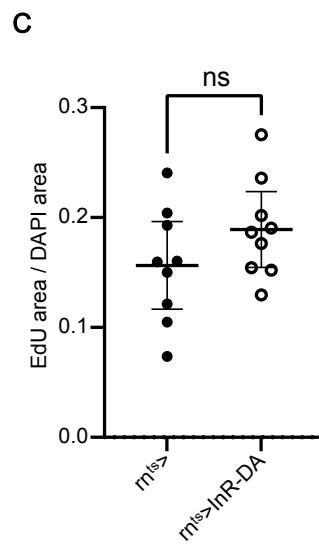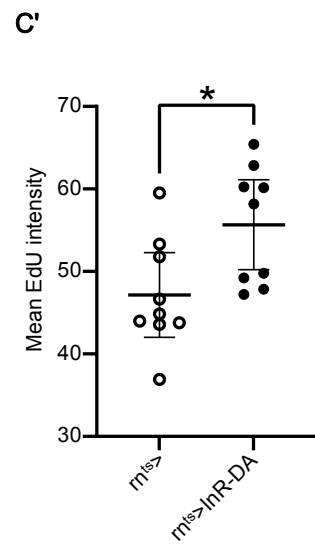

Supplement: S8 Fig — (A,B) Control wing disc (A), wing disc after 24 h of UAS-InR-DA expression in the pouch domain (B). Discs were stained with DAPI to visualize nuclei and were assessed for DNA replication activity by EdU incorporation. (C) Quantification of the percentage of DAPI areas that were positive for incorporated EdU in control wing discs or UAS-InR-DA expressing wing discs. This serves as a proxy for the number of nuclei undergoing DNA replication (WT, n = 9 discs, UAS-InR-DA, n = 9 discs, p = 0.1711). (C’) Quantification of incorporated EdU, measured as mean EdU intensity in the EdU area within the pouch. A Welch’s test was performed to test for statistical significance. (WT, n = 9 discs, UAS-InR-DA, n = 9 discs, *p = 0.0187). Single sections are shown in (A,B). Scale bars: 50 μm. (PDF) [file pgen.1010516.s008.pdf]
