# Supplementary material for: Distinct signaling signatures drive compensatory proliferation via S-phase acceleration
Source: PLoS Genet. 2022 Dec 15;18(12):e1010516. doi: 10.1371/journal.pgen.1010516 (PMC9799308; doi:10.1371/journal.pgen.1010516)
Supplement: S1 Table — (DOCX) [file pgen.1010516.s009.docx]

**Supplementary Table S1**

**Fly strains used in this study**

| Genotype | Source |
| --- | --- |
| *enGAL4, UAS-GFP* |  |
| *hep(R75)* | BDSC 6761 |
| *rn[GAL4-DeltaS], tubGAL80[ts]* |  |
| *rn[GAL4], UAS-egr, tubP-GAL80[ts]* | I. Hariharan |
| *UAS-bskDN* | BDSC 6409 |
| *UAS-GFP* | BDSC 1521 |
| *UAS-hid* | G. Morata |
| *Ubi-GFP.E2f1.1–230, Ubi mRFP1.NLS.CycB.1–266* | BDSC 55123 |
| *UAS-puc-RNAi* | BDSC 31557 |
| *TRE-RFP (attP40,II)* | D. Bohmann |
| *Act5C(FRT.polyA)lacZ.nls, ubi-p63E(FRT.STOP)GFP/CyO* | BDSC 51308 der. |
| *hsflp [122]; Sp/CyO, ubi-GFP; Dr/ TM6c* |  |
| *UAS-Mmp1.f2* | BDSC 58702 |
| *UAS-Mmp2/III* | BDSC 58706 |
| *UAS-cora-RNAi* | BDSC 51845 |
| *yki::GFP/CyO, kr-GFP* | B. Thomson |
| tub-miniCic::mCherry (II) | R. Levayer |
| *10XStat92E-DGFP / TM6C, Sb Tb* | BDSC 26200 |
| *UAS-Egfr.DN; UAS-Egfr.DN* | BDSC 5364 |
| *UAS-Ras^V12^ /TM6c* | BDSC 4847 |
| *UAS-yki.S168A.V5* | BDSC 28818 |
| *w; FRT42D yki (B5)/ CyO* | I. Hariharan |
| *UAS-hpo-RNAi* | VDRC 104169 |
| *UAS-wts-RNAi* | VDRC 111002 |
| *Pbac (Myc::GFP.FPTB)/TM6C, Sb* | BDSC 81274 |
| *UAS-Myc.HA (III)* | BDSC 64759 |
| *UAS-Stat92E-3xHA, (attP-86Fb)* | ORFeome Project |
| *FRT82B stat 92E [85c9] / TM6c* | E. Bach |
